# Biological applications of yttrium oxide nanocomposites synthesized from *Aspergillus penicillioides* and their potential role in environmental remediation

**DOI:** 10.1038/s41598-025-21104-4

**Published:** 2025-10-24

**Authors:** Yamini Vinayagam, Devi Rajeswari Vijayarangan

**Affiliations:** Department of Bio-Medical Sciences, School of Biosciences and Technology, Vellore Institute of Technology, Vellore, Tamil Nadu India

**Keywords:** *Aspergillus penicillioides*, Bioremediation, Fungal bioactive molecules, Industrial effluent, Toxic metals, Yttrium oxide nanoparticles, Biological techniques, Biotechnology, Microbiology, Environmental sciences, Nanoscience and technology

## Abstract

Industrial effluent contains hazardous metals, such as lead and nickel, which must be removed to prevent serious illnesses. The biological production of nanocomposites, which utilizes a range of biological resources, including microbes and plant extracts, as reducing and stabilizing agents, has garnered considerable attention in recent years. The goal of the current study is to generate Y_2_O_3_ nanocomposites in a unique, feasible, and biodegradable manner by using biological substances for the remediation of lead and nickel in the wastewater. The characterization outcomes demonstrated that *Aspergillus penicillioides* was capable of extracellular synthesis of Y_2_O_3_ nanocomposites with a spherical shape, and their size ranges from 31.4 to 61.9 nm. Furtherly, FTIR reveals that the existence of fungal biomolecules plays a vital role in the synthesis of the nanocomposite. The Y_2_O_3_ nanocomposites exhibited an excellent adsorption rate of 60% for lead and nickel at concentrations of 4 µg/mL and 2 µg/mL, respectively, under a pH of 6, thus demonstrating their efficacy as a catalyst in the photocatalytic reduction of these metals. The Freundlich adsorption isotherm validated multilayer adsorption on a heterogeneous surface with changing adsorption energies, whereas the constant parameter in the reaction was identified by pseudo-first-order kinetics. AAS is employed to determine the concentrations of desired metal ions. The biological applications, which include antibiotic activity, bacterial protein leakage, and antioxidant activity, were demonstrated. Biocompatibility of the Y_2_O_3_ nanocomposite was determined by the hemolytic assay. Ultimately, our study exhibits the novel bio-based *Aspergillus penicillioides*-arbitrated Y_2_O_3_ nanocomposites that work well as a bioremediating agent.

**Supplementary Information:**

The online version contains supplementary material available at 10.1038/s41598-025-21104-4.

## Introduction

The hazardous metal concentration of wastewater from industries is a serious concern as it can cause soil necrosis near streams and negatively impact animals living near disposal sites. Particularly in highly populated urban regions where wastewater is frequently utilized in agriculture, human exposure to polluted water is frequent. Considering the harmful effects of many industrial operations, including tanneries, pulp and paper mills, and batteries on aquatic ecosystems, mitigating pollution from metals is a global concern. Region-specific contamination rates differ, although heavy metal exposure is a global concern. According to reports, the main causes of heavy metal pollution in aquatic habitats are wastewater and industrial waste released into water bodies. The heavy metals adhere to particles, aggregate in rivers, and endanger the environment subsequently^1^.

Industrial discharges containing heavy metals, dyes, fertilizers, pesticides, and other chemical compounds introduce these contaminants into wastewater streams. The resulting soil contamination arises from two key factors: the stagnant, settled nature of wastewater itself, as well as the reuse of this contaminated wastewater for agricultural irrigation purposes^2^. Because of their greater density than water, metals are highly soluble in water and can attach themselves to the muscles and gills of fish. Because of their simple method of passing through the food chain, metal ions can be dangerous to living things. In humans, they can accumulate and cause serious health problems like cancer, renal failure, and cardiovascular illnesses^3^. The prevalence of heavy metals (HM) in the human body eventually results in toxicity, which in turn induces Minamata disease, Alzheimer’s disease, arrhythmia, muscular dystrophy, neurotrauma, multiple sclerosis, etc. Possibly immediate side effects such as vomiting, dehydration, sleepiness, nausea, renal failure, and stomach pain, or chronic side effects such as neurological disorders, physical abnormalities, muscular impacts, genetics, and hereditary difficulties can result from HM toxicity. The unrestrained exploitation of HMs to meet human needs has led to a critical level of contamination within the ecosystem. In living things, accumulation happens when certain metals are absorbed and stored at more rapid rates, followed by metabolization and excretion^4^.

Lead compounds, which comprise lead salts, oxides, and sulfides, includingdissolved lead, may be found in wastewater from industries. Lead can enter both aquatic and terrestrial environments when tainted wastewater is incorrectly dumped into rivers or sewage treatment plants. Lead may accumulate gradually in sediments, soils, plants, and animals, possibly leading to negative impacts on plant growth and contaminating water and other food sources^5^. In humans, even trace levels of lead in food or water can result in several health issues^6^, notably acute lead poisoning and persistent illnesses like cancer, diarrhea, nerve damage, paralysis, reduced cognitive function, and infertility. The permissible concentration of lead in agricultural soil is 85 ppm, but in wastewater, it is 0.01 ppm^5^.

Between 150,000 and 180,000 metric tonnes of nickel are released into the environment annually. Humans can be exposed to nickel compounds, including carbonyl, through inhaling aerosol particles, absorbing via the skin, or consuming tainted food or drink. Frequent exposure raises the amount of nickel in urine and increases the risk of allergies, skin infections, diarrhea, cancer, organ problems, and central nervous system malfunction. The acceptable limit of nickel in agricultural soil is 0.05 ppm, and in wastewater, it is 0.02 ppm^7^.

Nanobiotechnology employs a diverse array of methods that harness the capabilities of microorganisms and plants to synthesize beneficial nanocomposites composed of metals like calcium, titanium, gold, zinc, silver, yttrium, etc^8^. These metal oxide nanocomposites are expected to exhibit remarkable energy storage capabilities, therefore categorizing them as energy substances^9^. In medical, biomolecular detection, diagnostics^10^, medication administration, food production, agriculture, and waste management, among other fields, these nanocomposites are important^11^. Metallic nanocomposites have favorable optical, electrical, catalytic, magnetic, and biological properties, rendering them significant for applications in wastewater treatment, agriculture, tissue engineering, antibacterial, and antiviral approaches^12^. Yellow tartrazine (YT) dye is an environmental risk due to its harmful effects on fish and probable allergic reactions in people^13^. da Silva et al., 2019, reported that 48.7%, 50.2%, 35.3%, 40.7%, and 45.3% of imidazolium-based ions (C_4_MImPF_6_, C_4_MImCl, C_4_MMImNTf_2,_ C_4_MMImCl, & C_4_MImNTf_2_) were deteriorated under UV light, while 43.6%, 45.4%, 32.7%, 36.8%, and 40.2% of the same compounds were damaged under visible light, respectively. The commercial titania P25 catalyst reduced 50.2% and 66.3% of C_4_MImCl under visible and ultraviolet radiation, respectively^14^. The heterogeneous photocatalysis of Ag/TiNPs achieved destruction of RhB (k = 0.0219 min^− 1^) under visible light irradiation after 120 min, demonstrating potential for reuse across 5 cycles with only a 5% decrease in photocatalytic efficiency^15^.

Nanomaterials are an exceptionally effective, selective, and stable option for the aqueous removal of hazardous metal contaminants^16^. Trace elements are typically found in living tissues, and their shortage results in metabolic and structural imbalances^17^. Recent years have seen a surge in interest in yttrium oxide (Y_2_O_3_) as an important rare earth compound. In terms of chemical catalysis and optronics device design, it serves as one of the foremost potential components. The recognized properties of Y_2_O_3_ include a high dielectric constant and exceptional thermal stability when in the powdered condition. Functional composite substances such as yttria-stabilized zirconia films and extremely effective additives can be made from Y_2_O_3_. Furthermore, it is investigated for possible photodynamic treatment and biological imaging uses. It is also commonly utilized as a binding substance for several rare-earth doping agents^18^.

Yttrium oxide exhibits antioxidant activity under physiological conditions and is of substantial interest. According to a recent study, Y_2_O_3_ nanocomposites prepared with a biological substance-based scaffold can stimulate angiogenesis and vascularization, which may have implications for tissue engineering. Due to its ability to generate hydroxyl free radicals, which may adsorb a wide variety of dyes, photolysis is a possible substitute. Y_2_O_3_ nanocomposites exhibit significant luminescence efficacy and are ideal substrates for rare earth metals^19^.

The non-biodegradability of heavy metals renders the remediation of contaminated water and soil particularly difficult^20^. Conventional remedies have drawbacks about biological processes when applied on a large scale^21^. A diverse array of materials, including surfactant-enhanced activated carbon, iron-based soil amendments, mining products, and industrial residue and wastes, have been utilized for adsorption^9^. The primary advantages of the adsorption process compared to traditional treatment methods include lower cost, enhanced efficacy, less chemical usage, and the regeneration of the adsorbent post-reaction^22^. Biological agents are extensively utilized for the manufacture of metallic nanoparticles, encompassing both unicellular and multicellular species. Several significant examples include plant extracts, bacteria, viruses, fungi, yeast, algae, etc^23^. Fungi appear to be the most effective in heavy metal remediation due to their tolerance to stress conditions, rapid growth rate, and low nutritional requirements. Fungi can remove heavy metals through various techniques, including adsorption, ion exchange, valence transformation, intracellular precipitation, and extracellular precipitation. The fungal cell wall consists of chitin, a linear chain of beta-1,4-linked *N*-acetylglucosamine, together with polymers, glucan, proteins, and surface functional groups like amino, carboxyl, hydroxyl, phosphoryl, and imidazole^24^.

**Fig. 1:**
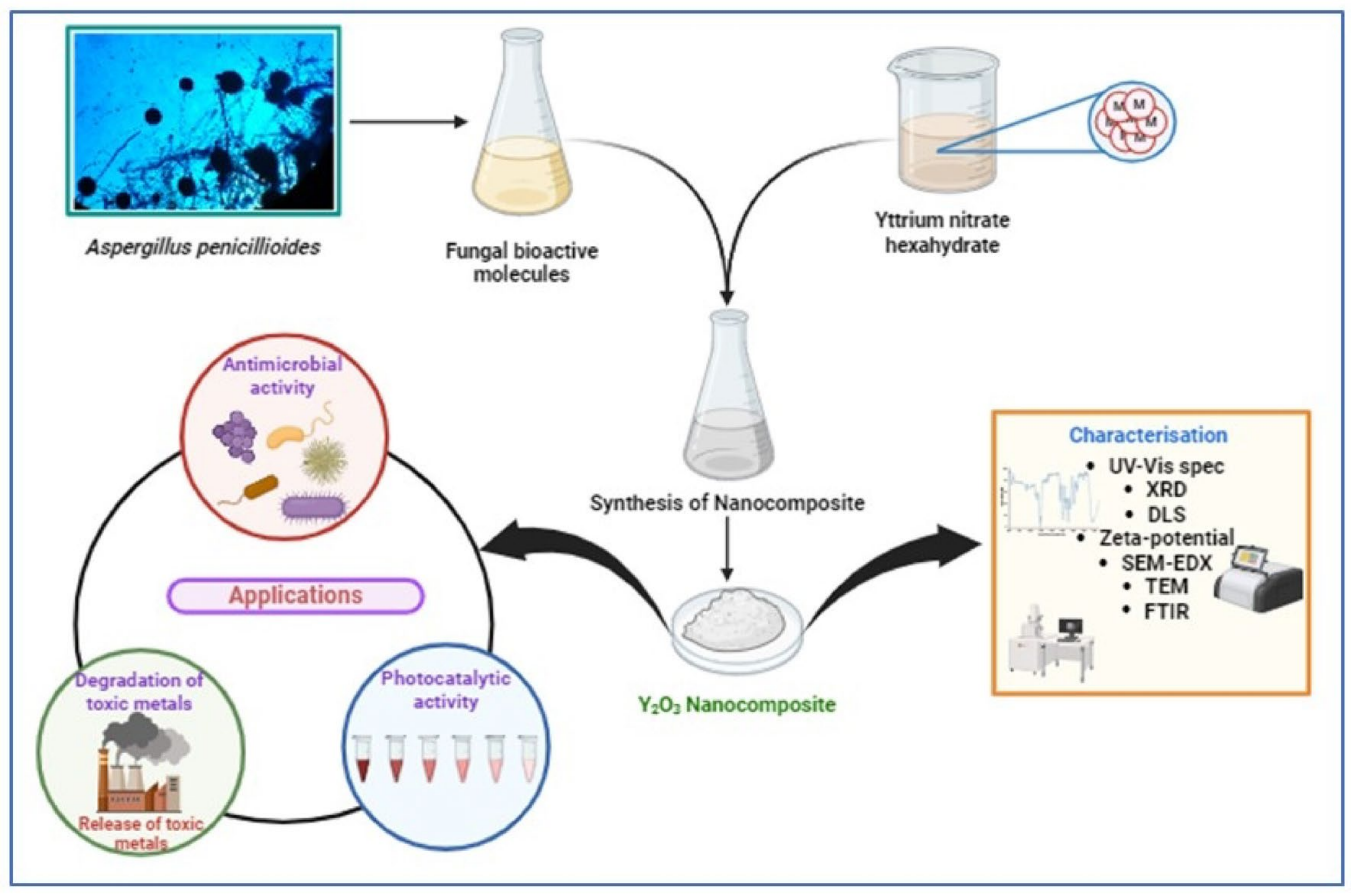
Graphical abstract of the researchstudy.

The primary goal of this research is to explore the adsorption process for removing lead and nickel from industrial wastewater, followed by the degradation of these toxic metals using Y_2_O_3_ nanocomposites synthesized with the aid of the fungus *Aspergillus penicillioides*. The biologically produced Y_2_O_3_ nanocomposites were thoroughly characterized, and the batch adsorption approach was employed to investigate the parameters affecting the adsorption process of lead and nickel metals^25^. Simultaneously, Y_2_O_3_ nanocomposites’ photocatalytic activity^26^ was investigated employing crystal violet dye. A quantitative technique called Atomic Absorption Spectroscopy was utilized to ascertain the quantity of hazardous metal ions, specifically deteriorated lead and nickel, present in the samples. Additionally, the biological applications have been demonstrated, including analysis of bacterial protein leakage, antioxidant properties^27^, and antibiotic efficacy^28^. The hemolytic assay was used to assess the Y_2_O_3_ nanocomposite’s ecological compatibility. This extensive approach investigates the extent to which *Aspergillus penicillioides* arbitrated Y_2_O_3_ nanocomposites eliminate the lead and nickel ions from wastewater from industries, offering insightful information for applications including environmental remediation (Fig. 1).

## Materials and methods

### Fungal isolation

Approximately 75 samples of soil and water from different locations in Tamil Nadu, India, were collected sterilely. Water samples were kept in sterile, airtight containers, whereas soil samples were collected with a sterile scalpel and sealed in sterile, closed bags. The samples were then kept at the ideal temperature. Numerous soil and water samples were utilized as inoculums for the serial dilution procedure, and they were plated in a potato dextrose agar (PDA) medium with 20 µg/mL of lead nitrate and nickel sulfate. Following 3 days of incubation at 25–27 °C, the growth plates were examined under a microscope to record microscopic findings and determine colony morphology. Subculturing in the PDA medium containing lead nitrate and nickel sulfate allowed for the selection and subsequent purification of specific fungal colonies. Eventually, the concentration of lead nitrate and nickel sulphate was gradually increased up to 1000 µg/ml, and the pure culture was maintained in the PDA slants. The fungal isolate was identified based on its morphological and microscopic attributes (Lactophenol cotton blue (LPCB) staining), including color, mycelia consistency, spore formation patterns, etc. The fungal genomic DNA was extracted by utilizing the chloroform-isoamyl alcohol method (EXpure Microbial DNA Isolation Kit). The DNA was then amplified using the primers ITS1 (5′ TCCGTAGGTGAACCTGCGC 3′) and ITS4 (5′ TCCTCCGCTTATTGATATGC 3′). The resulting PCR products were purified with the Montage PCR Cleanup Kit (Millipore) and sequenced using the ABI PRISM^®^ BigDyeTM Terminator Cycle Sequencing Kits in conjunction with AmpliTaq^®^ DNA polymerase (FS enzyme) (Applied Biosystems).

Moreover, single-pass sequencing was executed for every template employing universal primers for 18s rRNA. Through the process of ethanol precipitation, the fluorescently labeled fragments were isolated from the unregulated terminators. Following a resuspension in distilled water, the samples were electrophoresed using an ABI 3730xl sequencer (Applied Biosystems). The researchers employed the NCBI BLAST similarity search tool to conduct phylogenetic analysis, while the MUSCLE 3.7 software was utilized for generating multiple sequence alignments. Subsequently, the aligned sequences underwent refinement through the Gblocks 0.91b program, which eliminated aberrated regions and imperfectly aligned positions. After that, phylogenetic analysis was carried out utilizing the HKY85 substitution model and the PhyML 3.0 aLRT program. Using the Tree Dyn 198.3 program, an ensuing phylogenetic tree was generated.

### Extracellular biosynthesis of Y_2_O_3_ nanocomposites

The plant-associated fungus (*Aspergillus penicillioides*) was introduced into 200 mL of potato dextrose broth, which included lead nitrate and nickel sulfate. For 96 h, at a temperature of between 25 and 27 °C, the resulting mixture was incubated in a rotary shaker that operated at 130 rpm. Following the incubation phase, the culture medium underwent filtering through Whatmann’s No. 1 filter paper, and the fungal mycelia were subsequently disintegrated in 150 mL of autoclaved double-distilled water and shaken at a speed of 130 rpm for 24 h at room temperature. After this incubation period, the resulting suspension was filtered again using Whatmann’s No. 1 filter paper, and yttrium oxide (Y_2_O_3_) nanocomposites were made from the filtrate. Using a rotating evaporator, the impurities were eliminated, and the resulting mixture was allowed to evaporate until it was completely dry. Gas Chromatography and Mass Spectroscopy (GC-MS) analysis (Perkin Elmer Clarus 680 & 600) was used to further identify the metabolic compounds present in the *Aspergillus penicillioides* extract. A 50 mL fungal metabolite filtrate was combined with a similar amount of 0.1 M aqueous yttrium nitrate hexahydrate (Y(NO_3_)_3_.6H_2_O) to produce the yttrium oxide nanocomposites^29^. For 2 h, the resulting mixture was heated to 60 °C on a hot plate using a magnetic stirrer. Following the formation of a white deposit, the precipitate was extracted, dried, and calcined to create a fine granule that was subsequently employed in additional characterization techniques.

### Characterization studies on Y_2_O_3_ nanocomposites

Using an Agilent Technologies Cary 300 UV–visible spectrophotometer, the absorption of *Aspergillus penicillioides* arbitrated Y_2_O_3_ nanocomposites was measured in the 200–600 nm range. The nanocomposites underwent an examination of their optical characteristics through UV–visible spectrophotometer analysis. Additionally, the crystalline structure of the Y_2_O_3_ nanocomposites was identified by employing the X-ray diffraction (XRD) technique. The measurement was made on a Bruker D8 Advanced with a proportional counter and Cu Kα radiation (k = 1.5405 A, nickel filter) over a 2θ range of 10°–80° at a scanning rate of 5°/min. The Debye Scherrer equation, which can be expressed as follows (Eq. 1), is conceivably employed to determine the average crystalline grain size of the nanocomposite:

1 $${\text{D}}=\frac{{{\text{K}}\lambda }}{{\beta {\text{Cos}}\theta }}$$

Here, λ indicates the wavelength (1.54), β the entire width at half maximum, and K the Scherrer constant (0.98). D indicates the diameter of the crystalline nanocomposites. The lattice microstrain (ε) is calculated from the Eq. (2)^30^,

2 $${\text{Lattice}}\;{\text{microstrain}}=\frac{\beta }{{{\text{4Xtan}}\theta }}$$

The Dynamic Light Scattering (DLS) technique has been employed to determine the size distribution profile of synthesized nanocomposites in hydrodynamics. Zeta potential is the surface charge of the nanocomposites in a liquid solution. Zeta potential and DLS analysis may be performed with the Horbia Scientific SZ-100 equipment. It is an essential instrument that provides the condition of the surface of the nanocomposites and forecasts the colloidal dispersion’s long-term stability.

Scanning electron microscope (SEM) measurements of surface morphologies were conducted with the support of a SEM model: Hitachi S-3400 with 45KX amplification and running at a voltage of 15.0 kV. Using the Energy dispersive spectroscopy (EDS) technique, the elemental composition of Y_2_O_3_ nanocomposites was examined. TEM, or transmission electron microscopy (FEI – TECNAI; Model: G2–20 TWIN, operating voltage 200 kV), is an additional effective method for characterizing nanomaterials. The size, shape, and distribution of the particles can be ascertained quantitatively. Similar to SEM, TEM is an electronic spectroscopic imaging method, but with a better resolution. With Bruker FM: MIR – FIR/THz Spectroscopy, the presence of functional groups in Y_2_O_3_ nanocomposites was evaluated in the 4000–400 cm^− 1^ range (FTIR analysis). The absorption bands and spectral peak positions provide information about the synthesized Y_2_O_3_ nanocomposite’s chemical bonding, structure, and shape.

### Applications of the Y_2_O_3_ nanocomposites

#### Batch adsorption studies

The study evaluated the ability of the Y_2_O_3_ nanocomposite to adsorb lead and nickel (toxic metal ions) from simulated wastewater through a batch adsorption process driven by photocatalytic activity. The freshly generated wastewater containing lead nitrate and nickel sulfate (100 µg/mL) was mixed with 2 µL of Y_2_O_3_ nanocomposite (4 µg/mL) and swirled at room temperature at 150 rpm to evaluate the photocatalytic activity. Numerous procedures were optimized to obtain maximum adsorption, including pH (5–9), lead and nickel concentrations (2–100 µg/mL), Y_2_O_3_ nanocomposite concentrations (2–10 µg/mL), and the type of irradiation (UV and sunlight) for 5 h. Samples were removed from the adsorption study every 1 to 5 h on average. After everything was in balance, the catalyst was removed and centrifuged for 10 min at 8000 rpm to analyze it in a UV spectrometer. By using Eq. (3), the percentage of degradation was calculated.

3 $$\%\;{\text{of}}\;{\text{degradation=}}\frac{{({\text{Absorbance}}\;{\text{of}}\;{\text{control}}-{\text{Absorbance}}\;{\text{of}}\;{\text{sample}})}}{{{\text{Absorbance}}\;{\text{of}}\;{\text{control}}}} \times 100$$

#### Adsorption kinetics and isotherm studies for lead and nickel elimination

Adsorption isotherm and kinetics studies were employed to examine the rate, mechanism, propensity, and functions of the Y_2_O_3_ nanocomposite towards toxic metal ions such as lead and nickel adsorption. To assess the scientific information acquired in batch adsorption investigation, the Langmuir and the Freundlich isotherms, pseudo-first-order, and pseudo-second-order kinetics were examined^31^. Equations (4), (5), (6), and (7) showed the appropriate equations for each of them.

4 $$\frac{{{\text{C}}_{{\text{e}}} }}{{{\text{q}}_{{\text{e}}} }} = \frac{{{\text{C}}_{{\text{e}}} }}{{{\text{q}}_{{\text{m}}} }} + \frac{{\text{1}}}{{{\text{kq}}_{{\text{m}}} }}$$

5 $${\text{Log}}\;{{\text{q}}_{\text{e}}}=\frac{1}{{\text{n}}}\;{\text{log}}\;{\text{Ce}}\;{\text{+log}}\;{\text{k}}$$

6 $${\text{Log }}\left( {{\text{qe}} - {\text{qt}}} \right)={\text{log }}\left( {{{\text{q}}_{\text{e}}}} \right) - \frac{{\text{k}}}{{2.3}} \times {\text{t}}$$

7 $$\frac{{\text{t}}}{{{{\text{q}}_{\text{t}}}}}=\frac{1}{{{\text{kq}}{{\text{e}}^{\text{2}}}}}+\frac{{\text{t}}}{{{{\text{q}}_{\text{e}}}}}$$

#### AAS analysis of lead and nickel elimination

Lead and nickel were among the heavy metals that were identified using Perkin Elmer, AANALYST 400, atomic absorption spectroscopy (AAS). The hollow cathode lamps that used air acetylene as fuel were the source of radiation for Pb and Ni. Standard absorbances for Pb and Ni are 217 and 232, respectively, using a 0.7 slit width. Triplicate analyses were conducted on both initial and final concentrations of lead and nickel in the wastewater (i.e. after the water is treated with Y_2_O_3_ nanocomposites). The blank and standards were thus atomized, and the outcomes were noted. Following the plotting of a calibration curve for each solution, the sample solutions were atomized and examined. Employing the calibration in combination with the absorbance measurements performed for the unknown samples, the different metal concentrations in the samples were determined.

#### Photocatalytic activity of Y_2_O_3_ nanocomposite: dye degradation

To investigate the photocatalytic activity of *Aspergillus penicilliioides* arbitrated Y_2_O_3_ nanocomposites, the degradation of crystal violet (CV) under UV light was employed. The UV light source (365 nm) for this study consisted of 4 × 10 W bulbs. The reaction was set up using 0.1 g of Y_2_O_3_ nanocomposites in a container holding 10 mL of crystal violet solution at 10 ppm. The decomposition of the crystal violet has been observed using UV-vis spectroscopy, and the results suggest that Y_2_O_3_ nanocomposites might adsorb the crystal violet dye. Additionally, various concentrations of Y_2_O_3_ nanocomposites (50 µg/mL and 100 µg/mL) and time intervals (2 and 4 h) were used to investigate the photocatalytic abilities of the Y_2_O_3_ nanocomposites (2^3^ complete factorial design analysis)^32^.

#### Antibacterial study on Y_2_O_3_ nanocomposites

##### Agar-well diffusion method

The antibacterial characteristics of the synthesized *Aspergillus penicillioides* arbitrated Y_2_O_3_ nanocomposite have been evaluated using the agar well diffusion assay on the following bacterial strains: *Pseudomonas aeruginosa*,* Bacillus subtilis*, *Staphylococcus aureus*,* Escherichia coli*, and *Proteus vulgaris*^33^. Sterile swabs were used to swab the cultures of the strains specified above that were cultivated on Muller-Hinton agar plates. The agar plates were punctured and wells were filled with the Y_2_O_3_ nanocomposite at concentrations of 25, 50, 75, and 100 µg/mL. The plates were then incubated at 37 °C for 24 h hrs. The zone of inhibition values’ diameter indication was documented and examined with the positive control sample (ampicillin)^34^.

##### Bacterial growth kinetics

The bacterial isolates, including *P. aeruginosa*,* E. coli*,* P. vulgaris*,* S. aureus*, and *B. subtilis*, were cultured in LB broth and exposed to different concentrations of *Aspergillus penicillioides* arbitrated Y_2_O_3_ nanocomposites (50 and 100 µg/mL) to study their growth kinetics. The inoculated flasks were incubated at 37 °C in a rotary shaker for about 10 h to ensure proper agitation. The nanocomposites-treated bacterial cultures were used as samples, while untreated cultures served as controls. Optical density at 600 nm was measured hourly for up to 10 h, and these readings were plotted over time to evaluate the growth curves for the bacterial samples. The results allowed for the evaluation of Yttrium oxide nanocomposites’ effectiveness in inhibiting bacterial multiplication^35^.

##### Protein leakage analysis

The bacterial protein leakage assay was conducted using the Bradford assay. Gram-positive and Gram-negative bacteria, including *P. aeruginosa*,* E. coli*,* P. vulgaris*,* S. aureus*, and *B. subtilis*, were inoculated in sterile LB broth. Various concentrations (50 and 100 µg/mL) of myco-synthesized Y_2_O_3_ nanocomposites were added to the cultured flasks. Then, the flasks were incubated at 37 °C for 8 h, with controls maintained. Samples were centrifuged every 2 h at 6000 rpm for 15 min. Then, 200µL of the supernatant and 800µL of Bradford reagent were mixed and kept undisturbed at room temperature in the dark for 10 min. The absorbance of the samples was measured at 595 nm. Bovine serum albumin (BSA) was used as the standard protein^36^.

#### Antioxidant activity: DPPH assay

The 2,2-diphenylpicrylhydrazyl (DPPH) assay is a prominent biochemistry test used to evaluate a biomolecule’s ability to scavenge free radicals. To perform the assay, a 0.1 mM DPPH solution and various concentrations of Y_2_O_3_ nanocomposites were prepared using methanol. 3 mL of the DPPH reagent was mixed with the different concentrations of Y_2_O_3_ nanocomposites (20–100 µg/mL), properly combined, and incubated at room temperature for 30 min in the dark. The sample’s absorbance was then measured at 517 nm. Ascorbic acid was used as the standard, and 3 mL of DPPH in methanol served as the control^37^. The following formula (Eq. (8)) was used to calculate the DPPH scavenging potential:

8 $$\% {\text{ DPPH}}\;{\text{ radical }}\;{\text{scavenging }}\;{\text{assay}}=\frac{{\left( {{\text{Absorbance }}\;{\text{of}}\;{\text{ control }}-{\text{ Absorbance}}\;{\text{ of}}\;{\text{ sample}}} \right)}}{{{\text{Absorbance}}\;{\text{of}}\;{\text{control}}}}\; \times \;{\text{1}}00$$

#### Hemolytic assay

A hemolytic analysis was carried out to assess the cytotoxic effects of the myco-synthesized yttrium oxide nanocomposites in a biological system. This assay evaluated the immediate toxicity of the Y_2_O_3_ nanocomposite sample at different concentrations on human red blood cells (RBCs). To conduct the assay, 1 mL of heparinized human blood was collected from a donor, and the RBCs were isolated by centrifugationat 1000 rpm for 5 min. After removing the supernatant, the RBCs were washed with PBS. 100µL of 1% RBC suspension was incubated with different concentrations of Y_2_O_3_ nanocomposites (20, 40, 60, 80, and 100 µg/mL) for 2 h at 37 °C. Following incubation, the samples were spun at 1000 rpm for 5 min, and the supernatant’s absorbance was evaluated at 545 nm to assess RBC lysis. Triton X-100-treated RBCs were employed as a positive control, PBS-treated RBCs were utilized as a negative control, and ampicillin was employed as the standard^38^. The hemolysis percentage was determined using the formula (Eq. (9)):

9 $$\% \;{\text{of}}\;{\text{hemolysis}}=\frac{{({\text{Absorbance}}\;{\text{ of}}\;{\text{ sample}}-{\text{Absorbance }}\;{\text{of }}\;{\text{negative }}\;{\text{control}})}}{{\left( {{\text{Absorbance }}\;{\text{of}}\;{\text{ positive}}\;{\text{ control }} - {\text{ Absorbance}}\;{\text{ of}}\;{\text{ negative }}\;{\text{control}}} \right)}}\; \times \;{\text{1}}00$$

### Desorption and reutilization efficacy of Y_2_O_3_ nanocomposites

The adherence of lead and nickel to the surface of nanocomposites is a reversible process, allowing for the reutilization of nanocomposites following the desorption of metal ions. To extract metal ions from the nanocomposite surface, a 2.0 mL aliquot of a 1:1 combination of 0.1 mol/L NaOH and methanol exhibited optimal desorption efficiency. The adsorbent underwent many adsorption/desorption cycles to assess its reusability. Following each extraction, the adsorbent was employed in successive extractions after being eluted with the identical solution for the next cycle^39^. The adsorption-desorption experiment was conducted again, and Eq. (10) was utilized to calculate the adsorption efficiency.

10 $${\text{Adsorption}}\;{\text{efficiency }}\left( \% \right)=\frac{{{\text{V}}\left( {{{\text{C}}_{\text{o}}} - {{\text{C}}_{\text{e}}}} \right)}}{{\text{M}}}$$

In this context, C_o_ and C_e_ signify the initial and optimal metal ion concentrations (mg/L), respectively, V indicates the solution volume (L), and M denotes the mass of the Y_2_O_3_ nanocomposites (g).

### Statistical analysis

Each experiment was run through the process three times for each test, the standard deviation was computed, and the entire set of observations was subjected to one-way ANOVA, and p-value analysis was carried out by using Microsoft Excel and Minitab.

## Results and discussions

### Isolation and identification of fungal species by using 18s rRNA sequencing

**Fig. 2:**
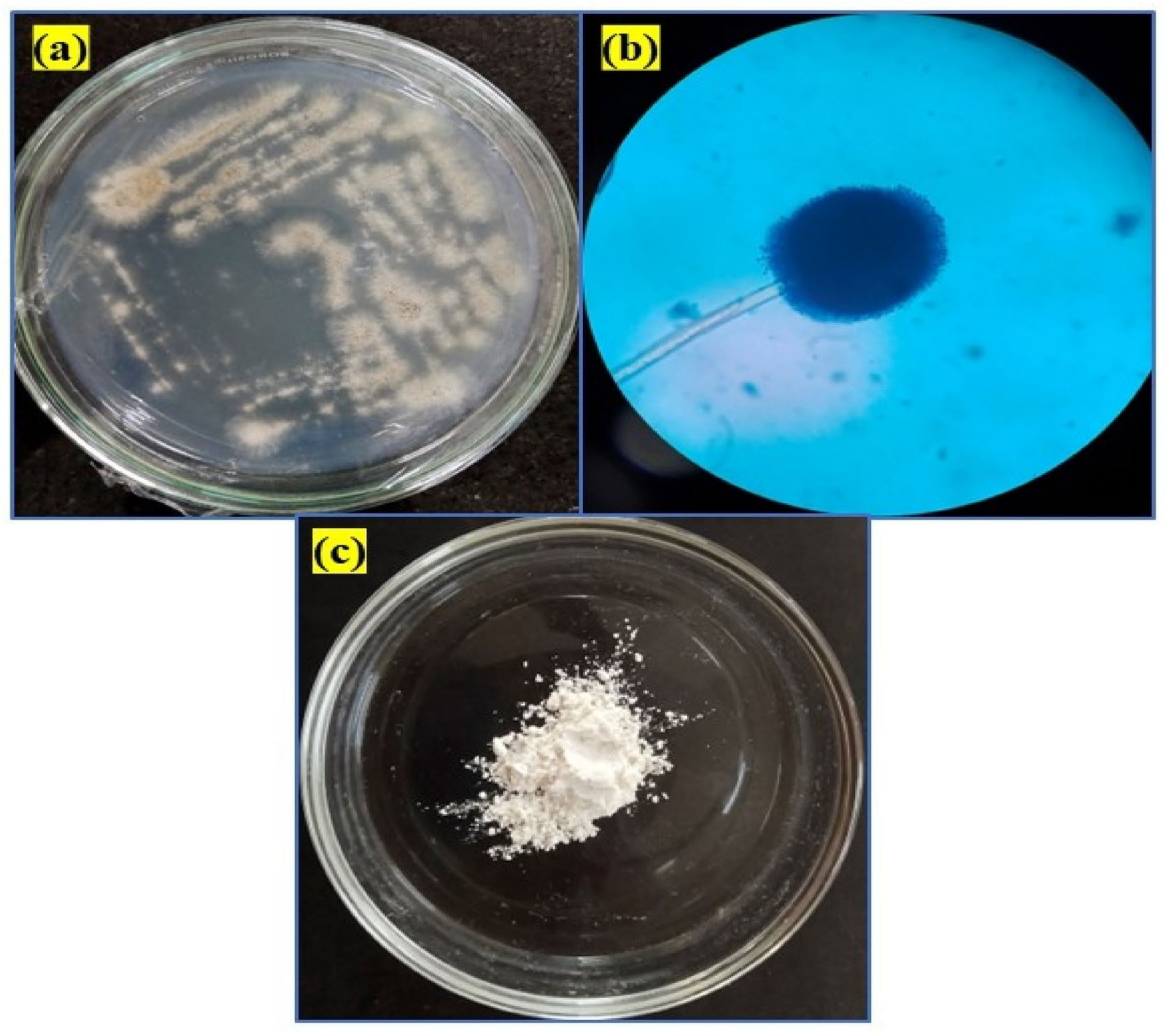
(**a**) The fungal colony initially appeared as white mycelium at the edges of the potato dextrose agar but rapidly turned black due to the production of conidia (asexual spores). (**b**) When stained with lactophenol cotton blue, the fungal hyphae (filaments) showed cross-walls (septa) and were transparent (hyaline). The conidial heads, which bear the conidia, were black and radiated outwards, tending to split into loose columns as they matured. The individual conidia were brown with irregular ridges, and the conidiophores (spore-bearing structures) had oval or flask-shaped vesicles. (**c**) The yttrium oxide nanocomposite material had a creamy white powder appearance.

**Fig. 3:**
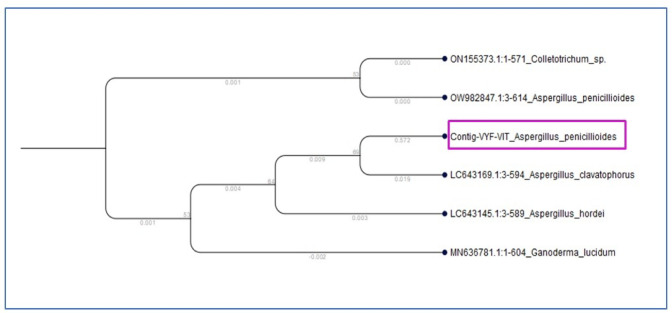
Phylogenetic tree for isolated fungal species.

The potato dextrose agar medium containing lead nitrate and nickel sulfate initially had white margins made of white mycelium, which swiftly turned black with conidial development (Fig. 2a). The reverse is white to cream in color. LPCB staining reveals septate and hyaline hyphae of the fungus. When conidial heads attain maturity, they begin to divide into loose columns and radiate in black color. Conidia have a brown color and sporadic ridges. Vesicles with an oval or flask shape are found in conidiophores (Fig. 2b). The isolated fungus’s genomic DNA was extracted via the chloroform-isoamyl alcohol method and subsequently amplified through PCR, utilizing the ITS1 forward and ITS4 reverse primers. In fungal species, the rRNA cistron region known as ITS1 is useful for metagenomic research. The ABI 3730xI sequencer was then used to acquire the ITS region sequencing, yielding 1210 base sequences. Additionally, the NCBI blast tool was used to blast the sequence, and MUSCLE 3.7 was used to perform the multiple sequence alignment. Additionally, phylogenetic analysis was performed using the program PhyML 3.0 aLRT, which determined that the isolated fungus was “*Aspergillus penicillioides*” (Fig. 3) and the gene sequence was documented in the NCBI gene sequence as ‘*Aspergillus penicillioides* VDRVYF’ with the accession number PQ570604.1 (https://www.ncbi.nlm.nih.gov/nuccore/2847402264).

A common food source of *Aspergillus penicillioides* is moisture-less foods such as grains, roasted nuts, fish, fruits, and herbs. In addition, it is commonly detected in shredded coconut, electronic meters, binocular glasses, and human skin. Various fungal isolates, including species from the genera *Aspergillus*,* Trichoderma*,* Fusarium*,* Phanerochaete*,* Rhizopus*, and *Pythium*, have demonstrated the potential to scavenge heavy metals. Among these isolates, *Aspergillus penicillioides* F12 (MN210327) manifested the ability to scavenge toxic metals such as lead (Pb) and cadmium (Cd) under optimal conditions of pH, temperature, and incubation time. The scavenging property of *Aspergillus penicillioides* F12 (MN210327) towards toxic metals like Pb, Cd, and Hg was found to be highest at optimal environmental conditions^40^.

### Extracellular biosynthesis of Y_2_O_3_ nanocomposites

**Fig. 4:**
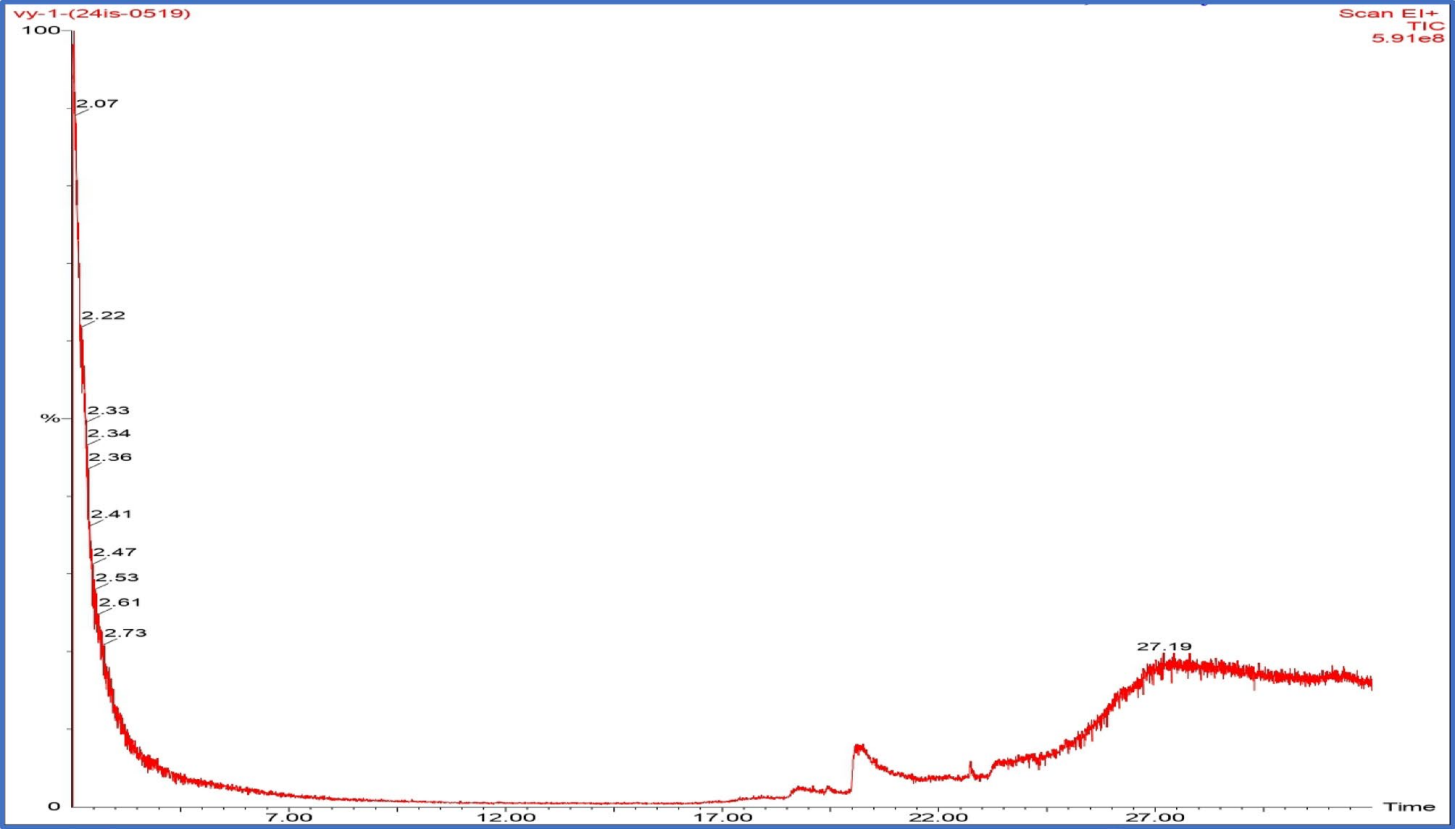
GC-MS analysis of *Aspergillus penicillioides* extract.

The bioactive molecules have been extracted and employed to produce Y_2_O_3_ nanocomposites using the extracellular synthesis process of *Aspergillus penicillioides*. GC-MS analysis was used to identify the metabolic components of *Aspergillus penicillioides*. The fungal extracellular extract chromatogram is displayed in Fig. 4. Eleven peaks were visible on the chromatogram, affirming the 2-to 30-min retention period. Utilizing the NIST library database, the metabolic compounds were identified^41^. The results of the mass spectrometry study, which confirm the presence of intermediate molecules and secondary metabolites, are shown in Table 1. The fungus extract yielded 20 compounds that were effectively determined. Potent production of Y_2_O_3_ nanocomposites results from the reaction of fungal bioactive compounds with yttrium nitrate hexahydrate (Y(NO_3_)_3_.6H_2_O), facilitated by an adequate stirring mechanism and regulation of temperature. Periodically monitoring the color change of the solution, it became creamy white, indicating the development of Y_2_O_3_ nanocomposites. The Y_2_O_3_ nanocomposites were white after the impurities were eliminated using the calcination process (600 °C for 3 h) (Fig. 2c). Bioactive molecules found in fungus extracts serve as capping, reducing, and stabilizing agents during nanocomposite formation.

**Table 1:** GC-MS analysis of *Aspergillus penicillioides* extract.

Sl.no	Name of the compound	Molecular mass (g/mol)	Molecular formula	Retention time
1	5-Dodecyne	166.3	C_12_H_22_	27.19
2	5-Dodecyne, 12-chloro-	200.75	C_12_H_21_Cl	27.19
3	6-Dodecyne	166.3	C_12_H_24_	27.19
4	Pentanal, 5-(methylenecyclopropyl)-	138.21	C_9_H_14_O	27.19
5	4-Acetoxy-2-azetidinone	129.11	C_5_H_7_NO_3_	2.61
6	Isoxazolidine, 4-ethyl-2,5-dimethyl-, cis-	129.2	C_7_H_15_NO	2.61
7	Methanamine, *N*-methyl-*N*-nitro-	90	C_2_H_6_N_2_O_2_	2.61
8	Propanedioic acid	104	C_3_H_4_O_4_	2.61
9	1 H-1,2,4-Triazole-3-carboxylic acid	113.08	C_3_H_3_N_3_O_2_	2.47
10	1,2,4-Triazole, 4-[*N*-(2-hydroxyethyl)-*N*-nitro]amino-	173.13	C_4_H_7_N_5_O_3_	2.33
11	1-Propanesulfonyl chloride	142.61	C_3_H_7_ClO_2_S	2.33
12	Hydrazine, 1-(5-hexenyl)-1-methyl-	128.22	C_7_H_16_N_2_	2.33
13	2-(2-hexyloxyethoxy)ethanol	190.28	C_10_H_22_O_3_	2.22
14	2 H-Pyran-2,5-diol, tetrahydro-, diacetate	202.21	C_9_H_14_O_5_	2.22
15	Acetoxy-(3-aminopropyl)butylborane	185	C_9_H_20_BNO_2_	2.22
16	cis-2,3-Epoxyoctane	128.21	C_8_H_16_O	2.22
17	1(2H)-Pyrazineacetonitrile, 5-amino-3,6-dihydro-3-imino-	151.17	C_6_H_9_N_5_	2.07
18	(R)-4,5-Dihydro-5-methyl-5-isoxazolecarboxylic acid methyl ester	143.14	C_6_H_9_NO_3_	2.07
19	Acetic acid, (3-allyloxy-1,1-dimethylbutyl) ester	200.28	C_11_H_20_O_3_	2.07
20	Pyrrolidine, 1,1′-methylenebis	154.25	C_9_H_18_N_2_	2.07

Organisms employ extracellular strategies to prevent the uptake of toxic metals. One of the initial extracellular defenses against metal toxicity in fungi is the binding or biosorption of metals. Other mechanisms to avert metal entry into cells include the extracellular release of enzymes that inhibit toxicants or agents that chelate pollutants, as well as the suppression of transporters that facilitate the influx of toxicants. Filamentous fungi are intriguing organisms for bioremediation applications due to their capacity to transfer molecules, including toxic metals, within their mycelium or midst of their mycelium and plant symbionts. Additionally, the microtubule system and secretory vesicles facilitate long-distance transport, serving as pathways and carriers for this translocation process^42^.

The extracellular production of Y_2_O_3_ nanocomposites is fundamentally linked to the heavy metal resistance pathways utilized by filamentous fungi in stressful environments. In response to toxic metal ions like Pb^2+^ and Ni^2+^, fungi activate adaptive mechanisms, which include the secretion of diverse extracellular bioactive compounds, including NADH-dependent nitrate reductase, acid phosphatases, thiol-containing peptides, metal-binding proteins, and low-molecular-weight chelators, including organic acids and phenolic compounds. These metabolites function as the primary defensive mechanism, obstructing metal ingress into the cytoplasm by complexing or decreasing ions in the extracellular environment. The release of metal not only safeguards the fungal cells but also generates a bioactive extracellular matrix that can diminish metal precursors and stabilize freshly synthesized nanocomposites^8^.

### Characterization of Y_2_O_3_ nanocomposites

**Fig. 5:**
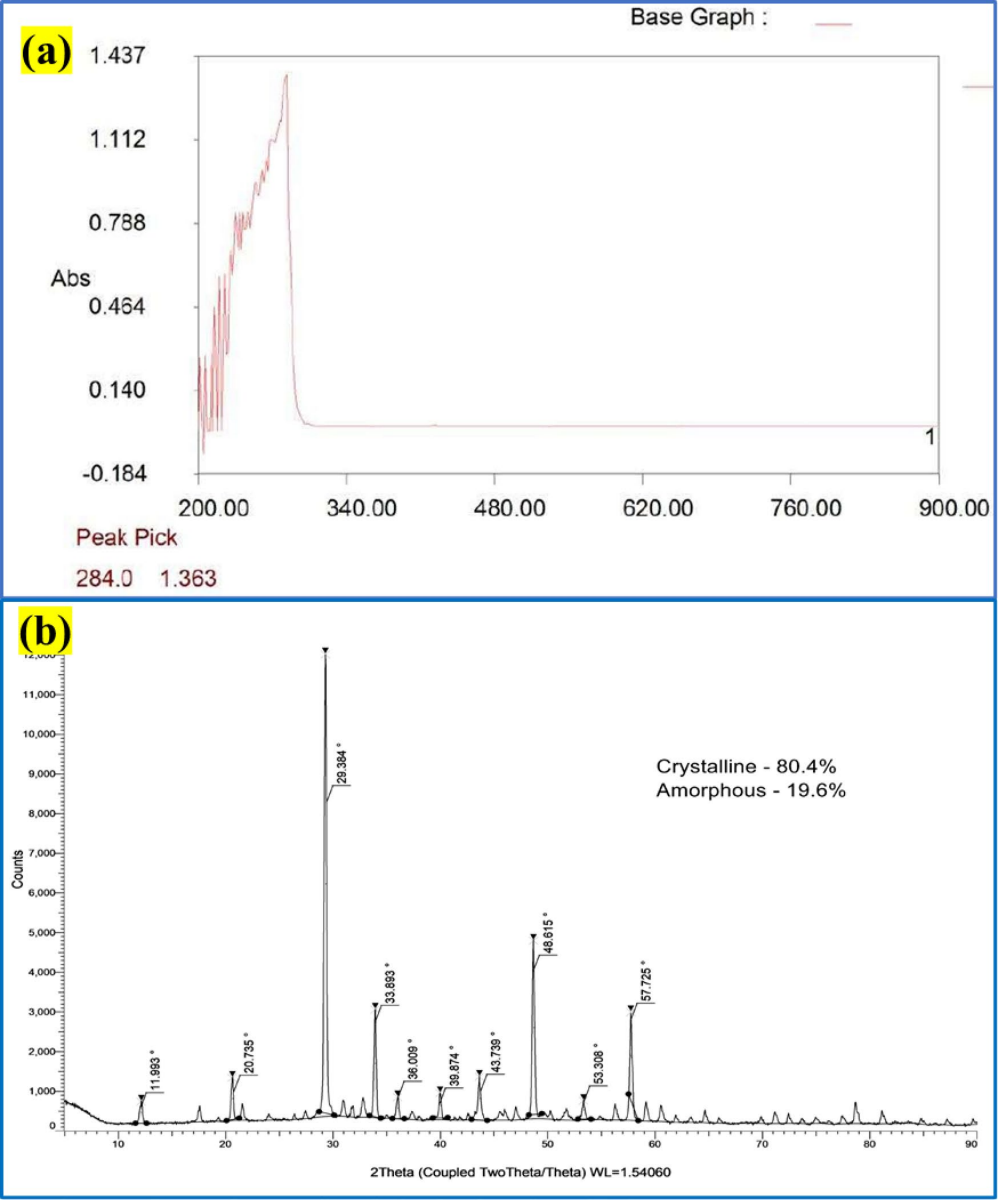
(**a**) UV–visible spectrophotometer of Y_2_O_3_ nanocomposites (**b**) XRD analysis of Y_2_O_3_ nanocomposites.

The UV-Vis spectrophotometer (Fig. 5a) provides insights into the optical energy band gap exhibited by the generated Y_2_O_3_ nanocomposites. A prominent and abrupt peak at 284 nm (λmax) is observed in the generated Y_2_O_3_ nanocomposites. This clarifies the electron excitation caused by photons absorbed from UV light from its valence to the conduction band. The optical energy band gap (Eg) of 4.37 eV has been computed. The absorption spectra of Y_2_O_3_ (yttrium oxide) typically exhibit an exciton band in the wavelength range of 235 to 400 nm. This characteristic band arises due to the inherent energy gap (bandgap), the midst of the valence band and the conduction band in the material. The bandgap results from the transfer of electrons through the valence band to the higher-energy conduction band^43^.

The X-ray diffraction analysis of the generated Y_2_O_3_ nanocomposites is displayed in Fig. 5b, with diffraction peaks located at 2θ values of 11.993, 20.735, 29.384, 33.893, 36.009, 39.874, 43.739, 48.615, 53.308, and 57.725. The XRD spectra of the nanocomposite material display multiple well-defined, intense peaks, indicating the presence of crystalline regions within the nanocomposite structure. The intense peaks at 2θ values of 29.384, 33.893, 43.739, 53.308, and 57.725 with corresponding planes (222), (400), (431), (440), and (622) show the thickness and lattice size of the generated Y_2_O_3_ nanocomposites. With a crystallinity of 80.4% and a lattice strain size of 0.00358, the synthesized Y_2_O_3_ nanocomposite’s typical crystal diameter was found to be 31.4 nm through the use of the Debye-Scherrer equation. The size, lattice thickness, and crystalline structure of the generated Y_2_O_3_ nanocomposites increase their likelihood of binding to lead and nickel molecules and boost their catalytic potential.

**Fig. 6:**
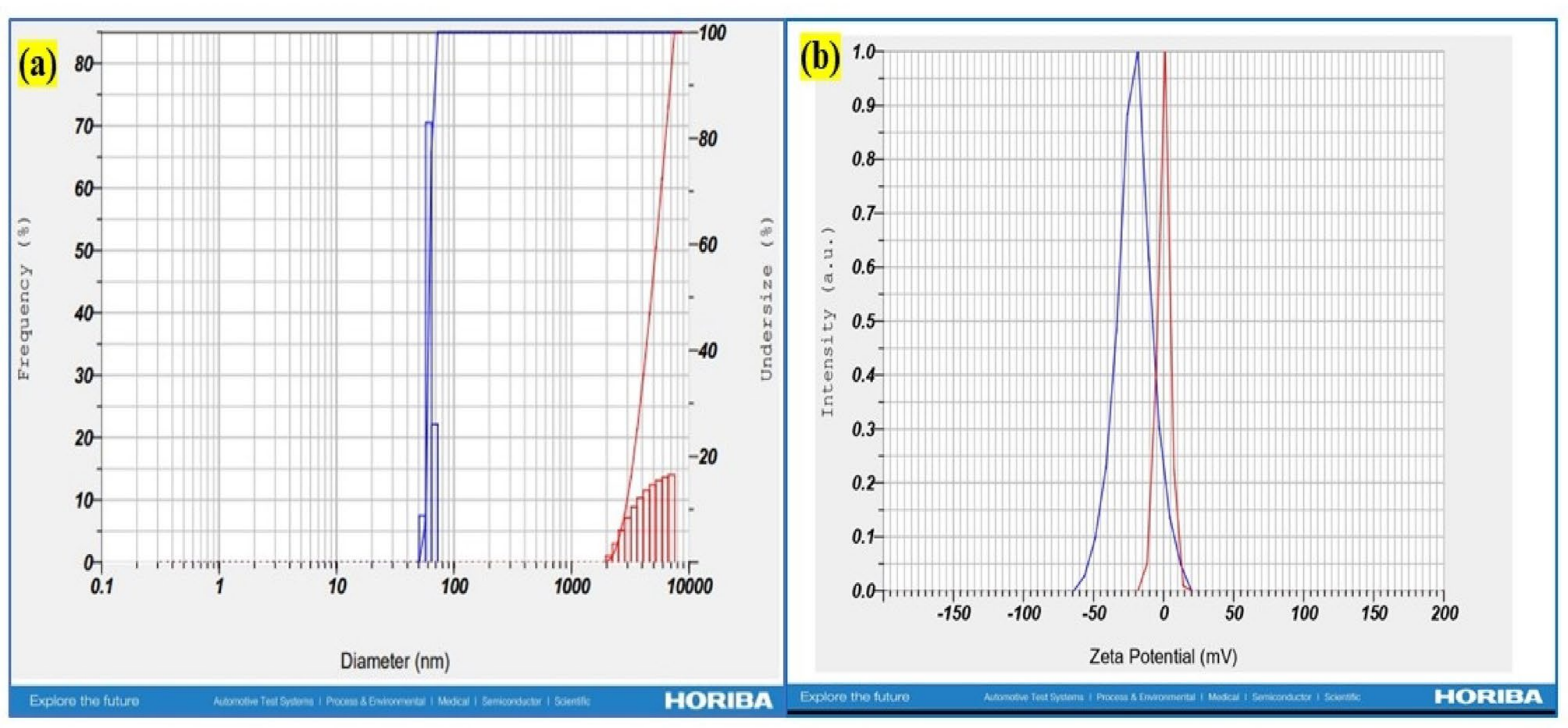
(**a**) DLS of Y_2_O_3_ nanocomposites (**b**) Zeta potential analysis of Y_2_O_3_ nanocomposites.

**Fig. 7:**
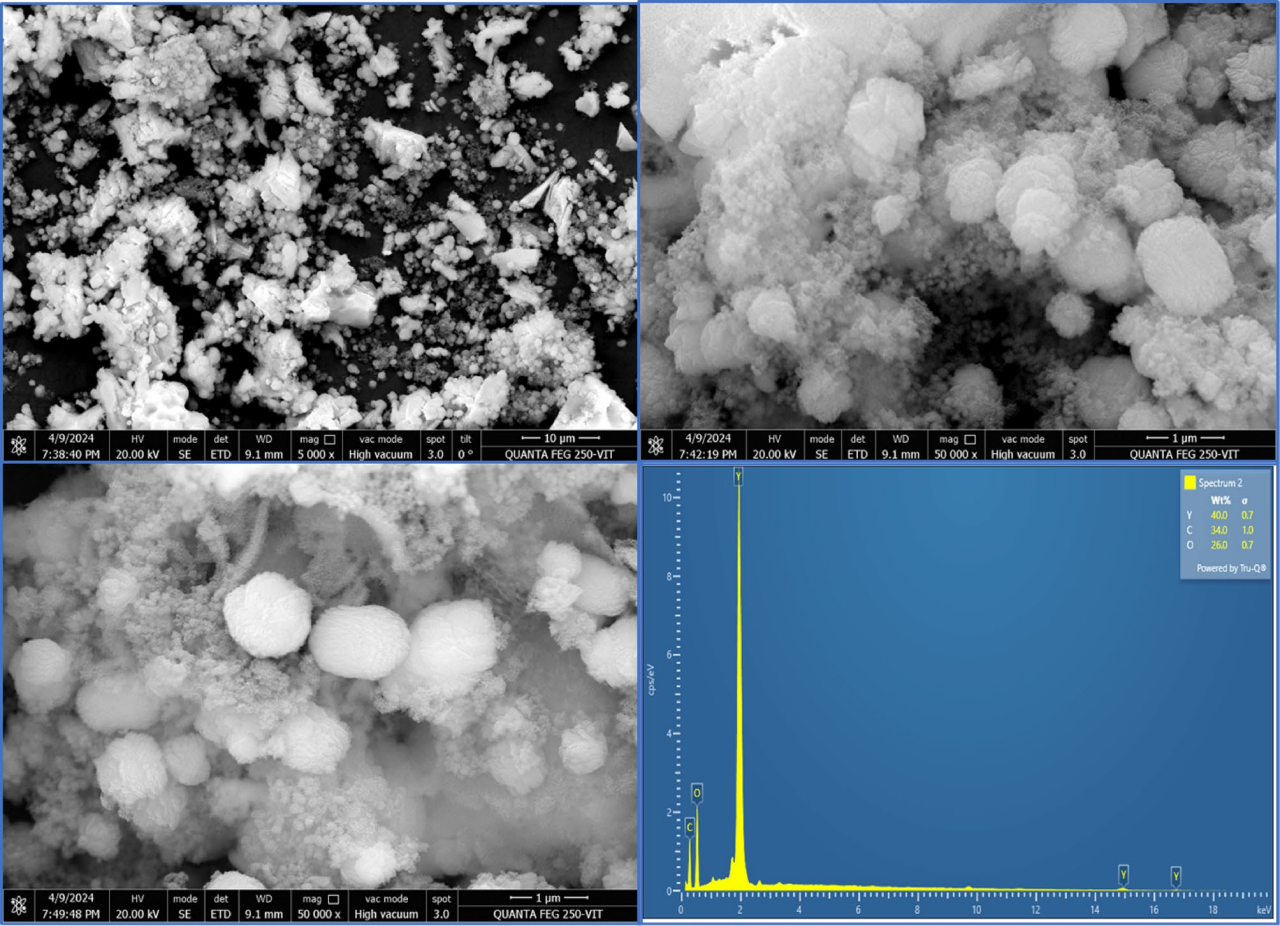
SEM-EDX study of Y_2_O_3_ nanocomposites.

**Fig. 8:**
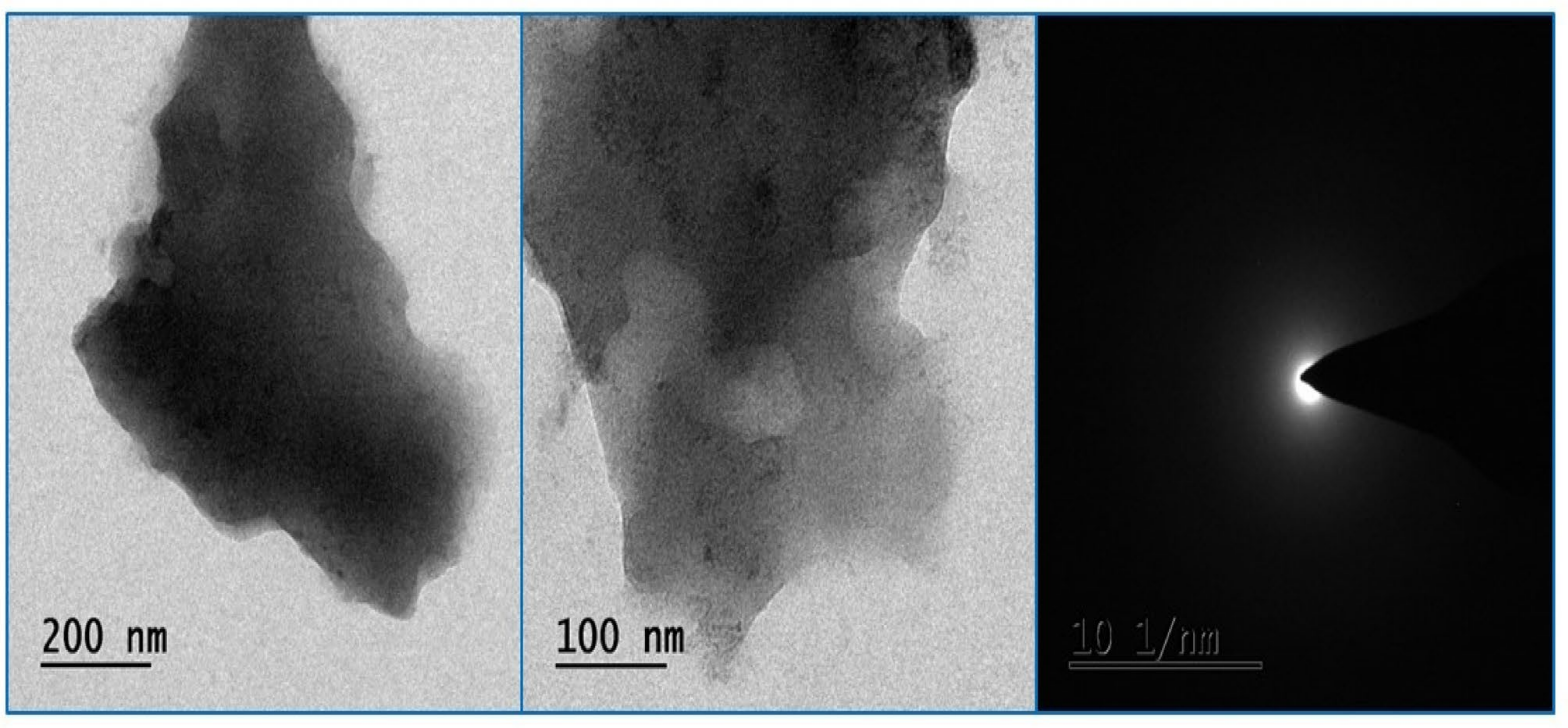
TEM analysis of Y_2_O_3_ nanocomposites.

The average particle diameter of the synthesized Y_2_O_3_ nanocomposites was 61.9 nm, as shown by the size distribution profile in Fig. 6a. By comparing XRD data with DLS data, it is confirmed that the generated nanocomposite ranges in size from 31.4 nm to 61.9 nm. As shown in Fig. 6b, the synthesized Y_2_O_3_ nanocomposites are thought to be a relatively stable colloidal dispersion, possessing adequate negative surface charge to hinder rapid aggregation, with a zeta potential of − 20.7 mV. Their anionic nature enhances colloidal stability and promotes electrostatic interactions with cationic heavy metal ions such as Pb^2+^ and Ni^2+^. SEM analysis reveals the morphology of the Y_2_O_3_ nanocomposites. Figure 7 displays a uniform spherical shape of the synthesized Y_2_O_3_ nanocomposites. Agglomeration is due to the high surface energy of the nanocomposites. According to the EDX spectrum analysis, Fig. 6 indicates the presence of C, O, and Y in atomic percentages of 57.75%, 33.10%, and 9.15%, respectively. Thus, the fabricated nanocomposites were highly pure, with the confirmation of the presence of yttrium. TEM analysis was used to execute a more thorough structural study. Figure 8 shows the entire surface of the Y_2_O_3_ nanocomposite at a lower magnification. An agglomeration of spherical particles characterized the morphology of the Y_2_O_3_ nanocomposite. Despite some of them being partially aggregated, the majority of the nanocomposites were organized well. TEM examination, on the other hand, shows almost uniformly sized sheet- and spherical-like particles. However, the TEM images also clearly show particle aggregation.

**Fig. 9:**
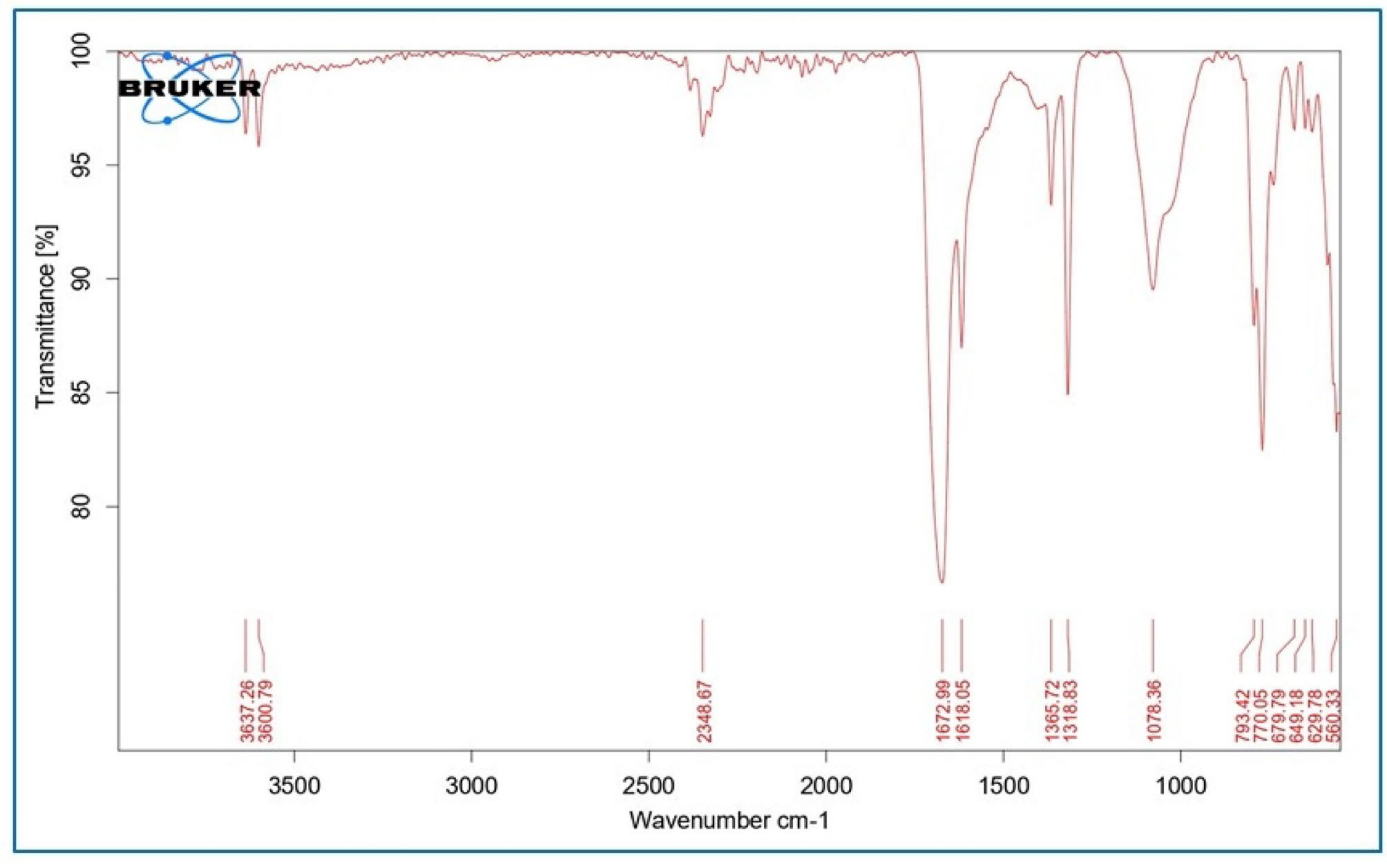
FTIR analysis of Y_2_O_3_ nanocomposites.

To assess the purity and composition of the Y_2_O_3_ nanocomposites, an FT-IR analysis was performed. The existence of any functional group or substance in the generated Y_2_O_3_ nanocomposites can be understood with the aid of the FT-IR spectra (Fig. 9). The broad absorption peaks at 3637 cm^− 1^, 3600 cm^− 1^, and 2348 cm^− 1^ are closely related to the hydrogen bond stretching vibration that occurs between the surrounding hydroxyl functional groups, the water molecules, and the presence of carbon on the surface. Additionally, the formation of peaks at 1078 cm^− 1^ and 679 cm^− 1^ indicates the C–O stretching and bending secondary alcohol group, whereas the broad and sharp peaks at 1365 cm^− 1^ and 770 cm^− 1^ are attributed to the C–H bending alkane and alkene group. The formation of sharp peaks at 1318 cm^− 1^ and 560 cm^− 1^ because to the linkage of S=O sulfone and Y-O stretching bond between the yttrium oxide and fungal bioactive molecules. Further, 1672 cm^− 1^ and 1681 cm^− 1^ attribute the C=O conjugated, C=C α, β-unsaturated ketone group ^32,44^. Moreover, the Y-O stretching peak at 560 cm^− 1^ specifies the yttria phase formation.

### Applications of the Y_2_O_3_ nanocomposites

#### Batch adsorption studies

**Fig. 10:**
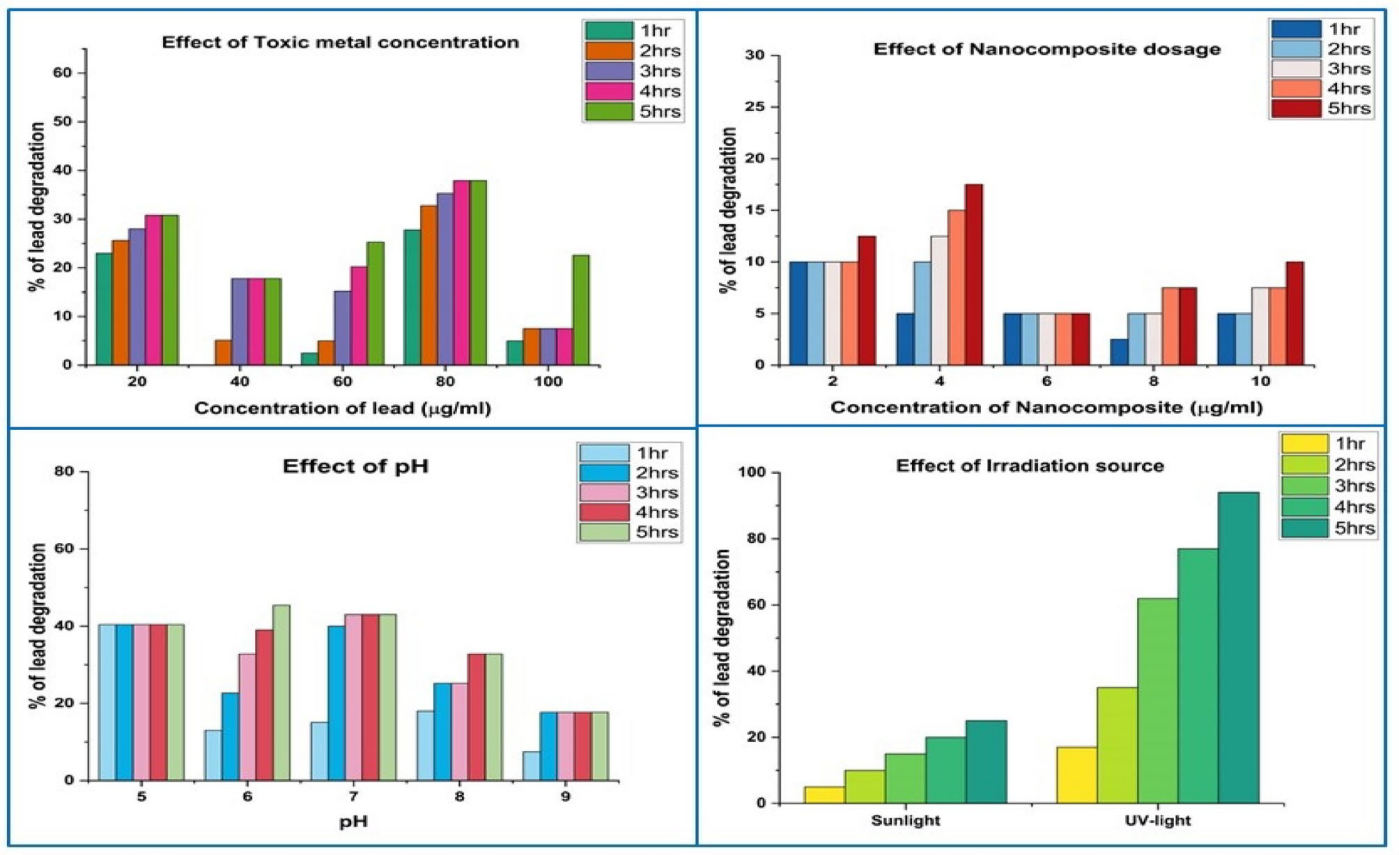
Batch adsorption studies Y_2_O_3_ nanocomposite on lead degradation.

**Fig. 11:**
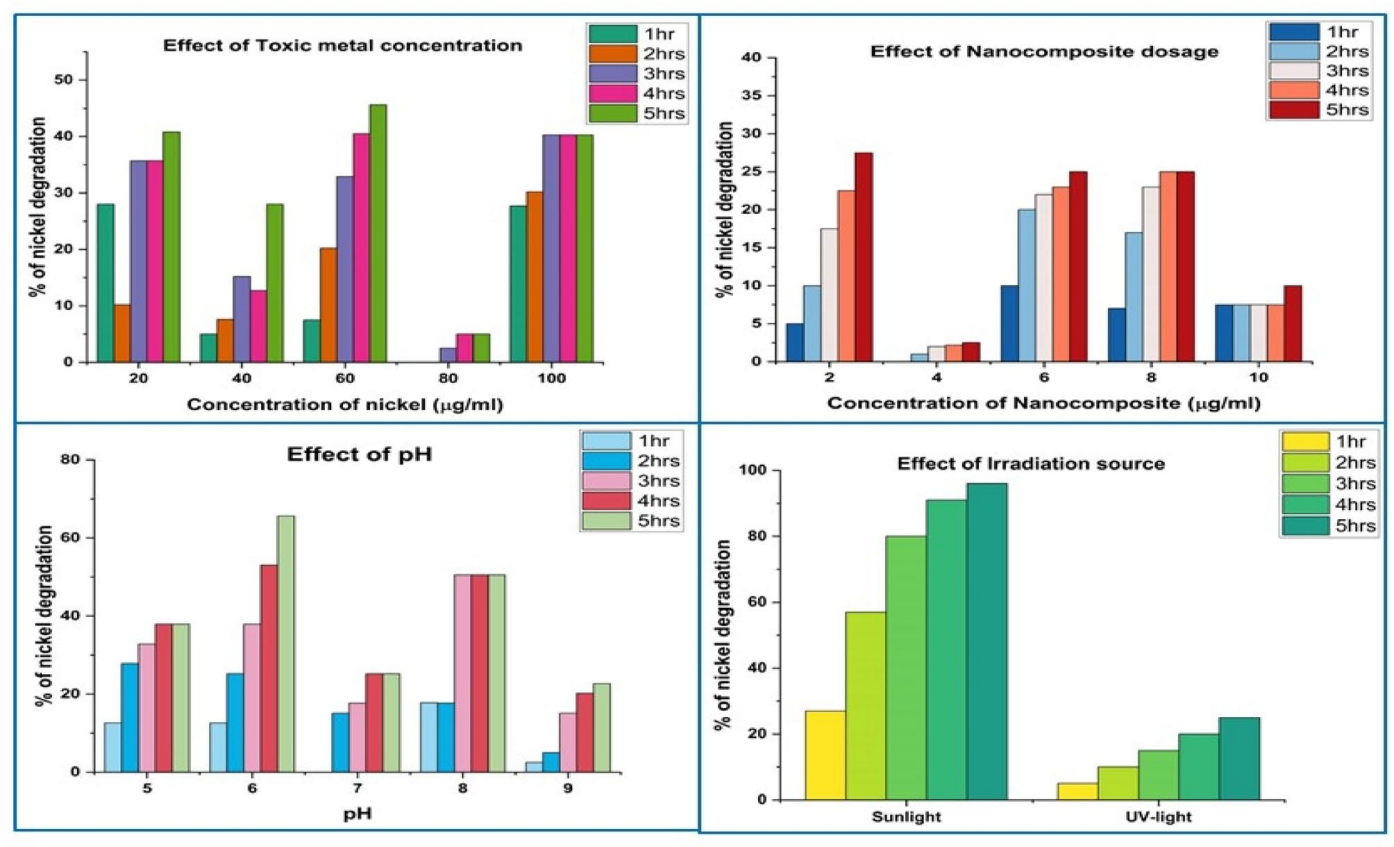
Batch adsorption studies Y_2_O_3_ nanocomposite on nickel degradation.

The effects of pH, the source of irradiation, the dosage of catalyst, the dosage concentration of lead and nickel (toxic metal ions), and the amount of time of stirring were studied for 1 to 5 h using Y_2_O_3_ nanocomposite to serve as the catalyst. The findings from the batch adsorption investigations suggest that the ideal conditions for lead removal are 60 µg/ml of lead and 4 µg/ml of Y_2_O_3_ nanocomposite in a reaction solution that is agitated for 5 h (Fig. 10). In Fig. 11, it is indicated that the pH of the solution is altered to 6 when exposed to a UV light source. A reaction mixture containing 60 µg/ml of nickel and 2 µg/ml of Y_2_O_3_ nanocomposite, with a pH of 6 and an irradiation source of UV light, was utilized to eliminate nickel. The degrading process for lead and nickel emerged after a comparison between the obtained parameters and the results.

Lead and nickel removal is reduced when both the amount of toxic metals and time increase. At a lead concentration of 60 µg/ml, the mixture must be agitated for 5 h to remove 60% of the lead at the maximum level. At lower concentrations, lead molecules bind to the active site of the Y_2_O_3_ nanocomposite. Since more active sites in the Y_2_O_3_ nanocomposite are occupied, lead reduction is hence reduced. Similarly, 70% of the nickel was adsorbed in 5 h at the maximum level of 60 µg/ml of nickel content.

Increasing the dosage of the adsorbent causes the toxic metals (lead and nickel) to be removed, and the removal level rises with time. Y_2_O_3_ nanocomposite concentration increases cause an increase in the amount of hazardous metal ions (lead and nickel) decomposition because more active sites are available for lead and nickel ions to adsorb. With an increase in the contact period between the hazardous metal ions and the nanocomposite, more lead and nickel ions promote up on the open sites of the Y_2_O_3_ nanocomposites. 70% of the lead and 75% of nickel may be efficiently removed in 5 h at a pH of 6, which is the lowest. When the pH of the mixture is 5, 7, 8, or 9, the removal percentage of toxic metal ions is lower. The lead and nickel ions adsorption and protonation of the Y_2_O_3_ nanocomposite were carried out at pH 6. The method of eliminating lead and nickel ions using a Y_2_O_3_ nanocomposite as a catalyst was conducted under UV light and sunlight as an irradiation source, with sunlight achieving maximum degradation of 23% of lead and 30% of nickel.

#### Adsorption isotherm and kinetics investigation

**Fig. 12:**
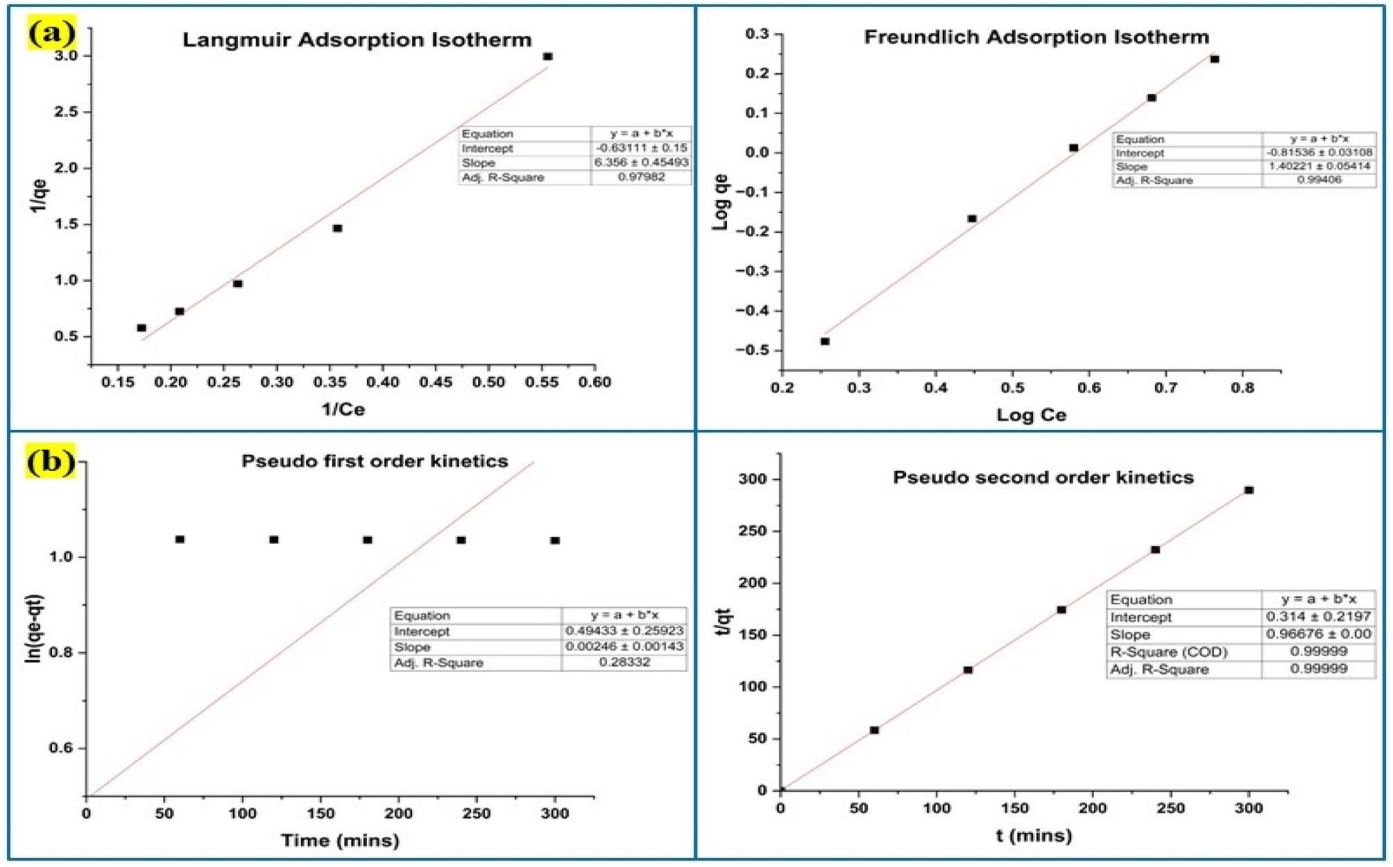
(**a**) Adsorption isotherm model for the adsorption of lead by Y_2_O_3_ nanocomposite (**b**) Adsorption kinetic model for the adsorption of lead by Y_2_O_3_ nanocomposite.

**Fig. 13:**
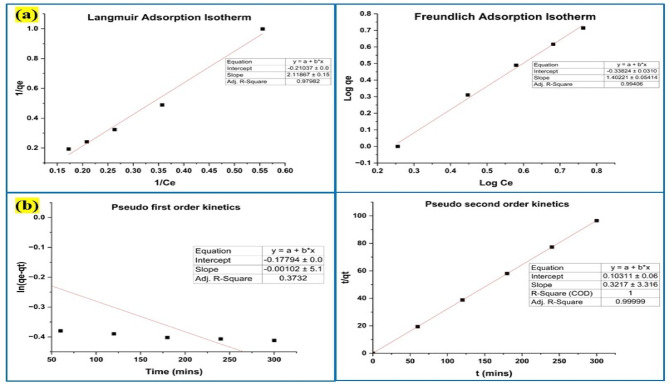
(**a**) Adsorption isotherm model for the adsorption of nickel by Y_2_O_3_ nanocomposite (**b**) Adsorption kinetic model for the adsorption of nickel by Y_2_O_3_ nanocomposite.

The validity of the data gathered from batch adsorption research was confirmed through analysis of the reaction mixture, which included the lead and nickel elimination product, for the adsorption isotherm and kinetic models. The Langmuir isotherm was expressed as C_e_ versus C_e_/q_e_, compared to the log C_e_ versus log q_e_ used in the Freundlich isotherm model^31^ as shown in Figs. 12a and 13a. Using the plots of pseudo-first-order (t vs. log (q_e_ − q_t_)) and pseudo-second-order (t vs. t/q_t_) kinetics variables^31^ for the lead and nickel adsorption results, were determined as well in Figs. 12b and 13b. Since hazardous metal ions’ adsorption on their surface was proven to fit both the pseudo-first-order kinetic and the Freundlich adsorption isotherm, the R^2^ values demonstrated the effectiveness of Y_2_O_3_ nanocomposites in the elimination of lead and nickel. The Freundlich adsorption isotherm validated multilayer adsorption on a heterogeneous surface with changing adsorption energies, whereas the constant parameter in the reaction was identified as pH by pseudo-first-order kinetics.

#### AAS analysis of lead and nickel elimination

The lead and nickel blank and standard samples begin by being used to calibrate the instrument. Atomic absorption spectroscopy was used to assess the lead and nickel (toxic metal ions) initial and final concentrations (treated with Y_2_O_3_ nanocomposites) in the wastewater. The final concentration of lead (1.172 mg/mL) and nickel (1.172 mg/mL) was found to be less than the initial level of concentration of toxic metal ions (lead (0.80 mg/mL) & nickel (0.736 mg/mL)) (Table 2) and it indicates the adsorption percentage of lead and nickel is 31.74 and 37.2. Consequently, the produced Y_2_O_3_ nanocomposites serve as an efficient bioremediating agent and are suitable for the treatment of wastewater from industries.

**Table 2:** AAS analysis for lead and nickel degradation by using Y_2_O_3_ nanocomposite.

Sample name	Pb	Ni
Initial concentration (S1)	1.172	1.172
Final concentration (S2)	0.80	0.736

#### Dye degradation: factorial design statistical analysis

As shown in Table 3, many factors, including pH, concentration, and time, were investigated at both the lowest (pH is 2; concentration is 50 µg/mL, and time is 2 h) and highest values (pH is 8; concentration is 100 µg/mL, and time is 4 h). The percentage of dye degradation was examined in the eight trials for each of the three parameters shown in Table 4. Using + 1 and − 1 for high and low levels, accordingly, an array was developed based on the analysis of 2^3^ complete factorial designs with 8 tests for the elimination of crystal violet. The encoded values of the parameters and the outcomes (% elimination efficiency). More than 94% of dye degradation was observed in the pH of 4 & 8, Concentrations of 50 & 100 µg/mL, and a time of 4 h. The results confirm that time plays a key role in the dye degradation. The associations among the independent variables were evaluated by employing ANOVA (Analysis of Variance), and the fundamental impacts of crystal violet dye adsorption were selected following the results that were greater than a 95% confidence level^45^.

**Table 3:** The different factors affecting dye degradation.

Factors	High level	Low level
Time	4	2
pH	8	2
Concentration	100	50

**Table 4:** The factorial design of 2^3^ shows 8 tests for dye degradation.

Runs	pH	Y_2_O_3_ nanocomposite (µg/mL)	Time (h)	% of dye degradation
1	1(8)	1(100)	1(4)	97.7
2	− 1(2)	1(100)	1(4)	77.5
3	− 1(2)	− 1(50)	1(4)	71.1
4	1(8)	1(100)	− 1(2)	12.5
5	− 1(2)	− 1(50)	− 1(2)	21.9
6	1(8)	− 1(50)	1(4)	94.2
7	1(8)	− 1(50)	− 1(2)	6
8	− 1(2)	1(100)	− 1(2)	29.8

The coded equation: Y = predicted response (% removal efficiency), X_0_ = global mean, X_1_–X_3_ = regression coefficients corresponding to impacts of primary parameters (time, pH, and Y_2_O_3_ concentration in the initial solution), and X_4_ = interaction terms, delineated the 2^3^ factorial analysis of crystal violet elimination by Y_2_O_3_ nanocomposites.

$${\mathbf{Y}}\,=\,{{\mathbf{X}}_{\mathbf{0}}}\,+\,{{\mathbf{X}}_{\mathbf{1}}}{\mathbf{A}}\,+\,{{\mathbf{X}}_{\mathbf{2}}}{\mathbf{B}}+{{\mathbf{X}}_{\mathbf{3}}}{\mathbf{C}}+{{\mathbf{X}}_{\mathbf{4}}}{\mathbf{AB}}+{{\mathbf{X}}_{\mathbf{5}}}{\mathbf{AC}}+{{\mathbf{X}}_{\mathbf{6}}}{\mathbf{BC}}+{{\mathbf{X}}_{\mathbf{7}}}{\mathbf{ABC}}$$

$$\% \;{\text{of}}\;{\text{ dye}}\;{\text{ degradation}}\,=\,{\text{12}}.{\text{17}}-{\text{1}}0.{\text{97 pH}}\,+\,{\text{8}}.{\text{93 time}}\,+\,0.{\text{1215 Concentration}}\,+\,{\text{3}}.{\text{825 pH}}*{\text{Time}}$$

**Fig. 14:**
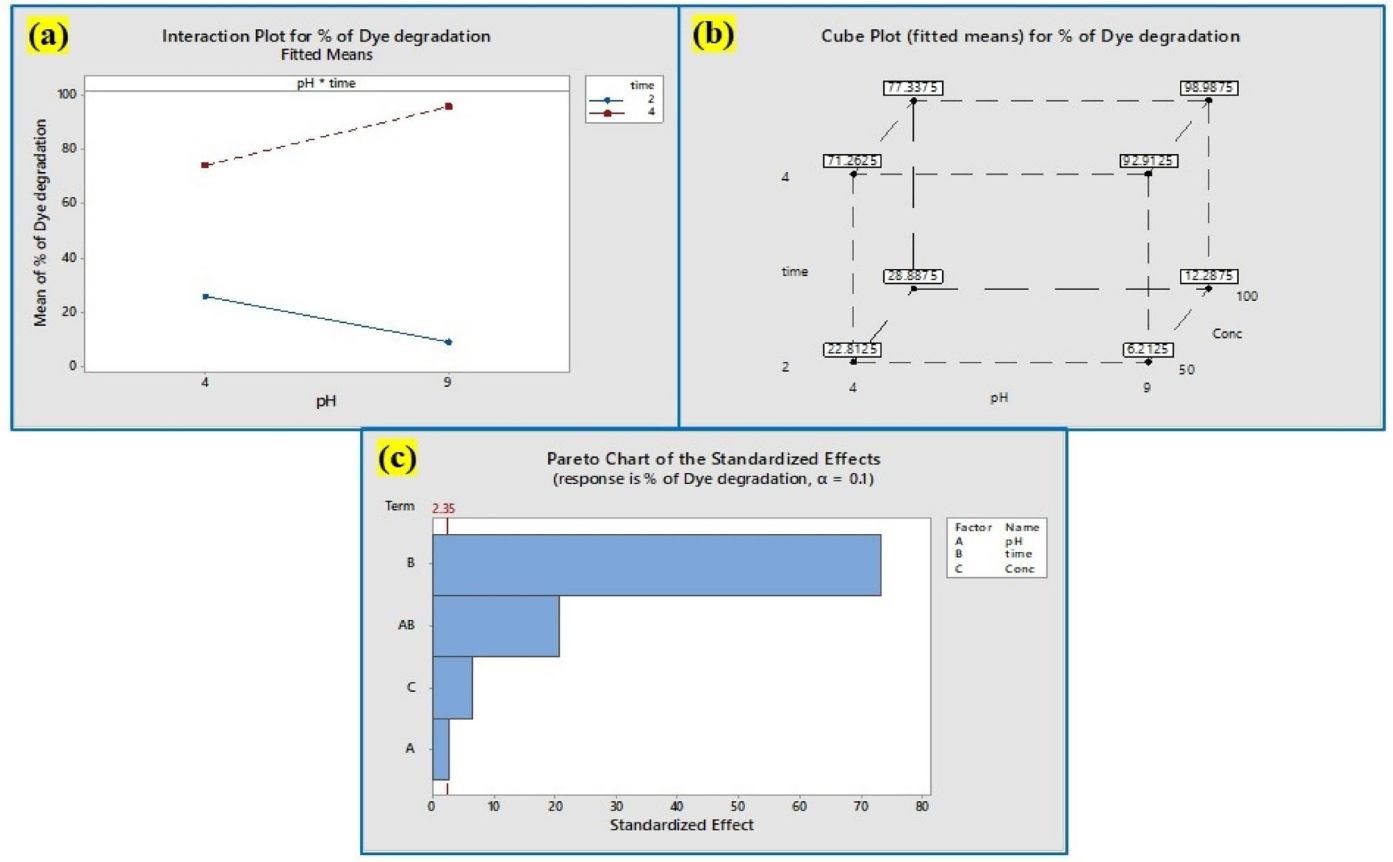
(**a**) Interaction plots for % of dye degradation (**b**) Cube plots for % of dye degradation (**c**) Pareto Charts showing the effect of pH, time, and concentration on the % of dye degradation.

**Fig. 15:**
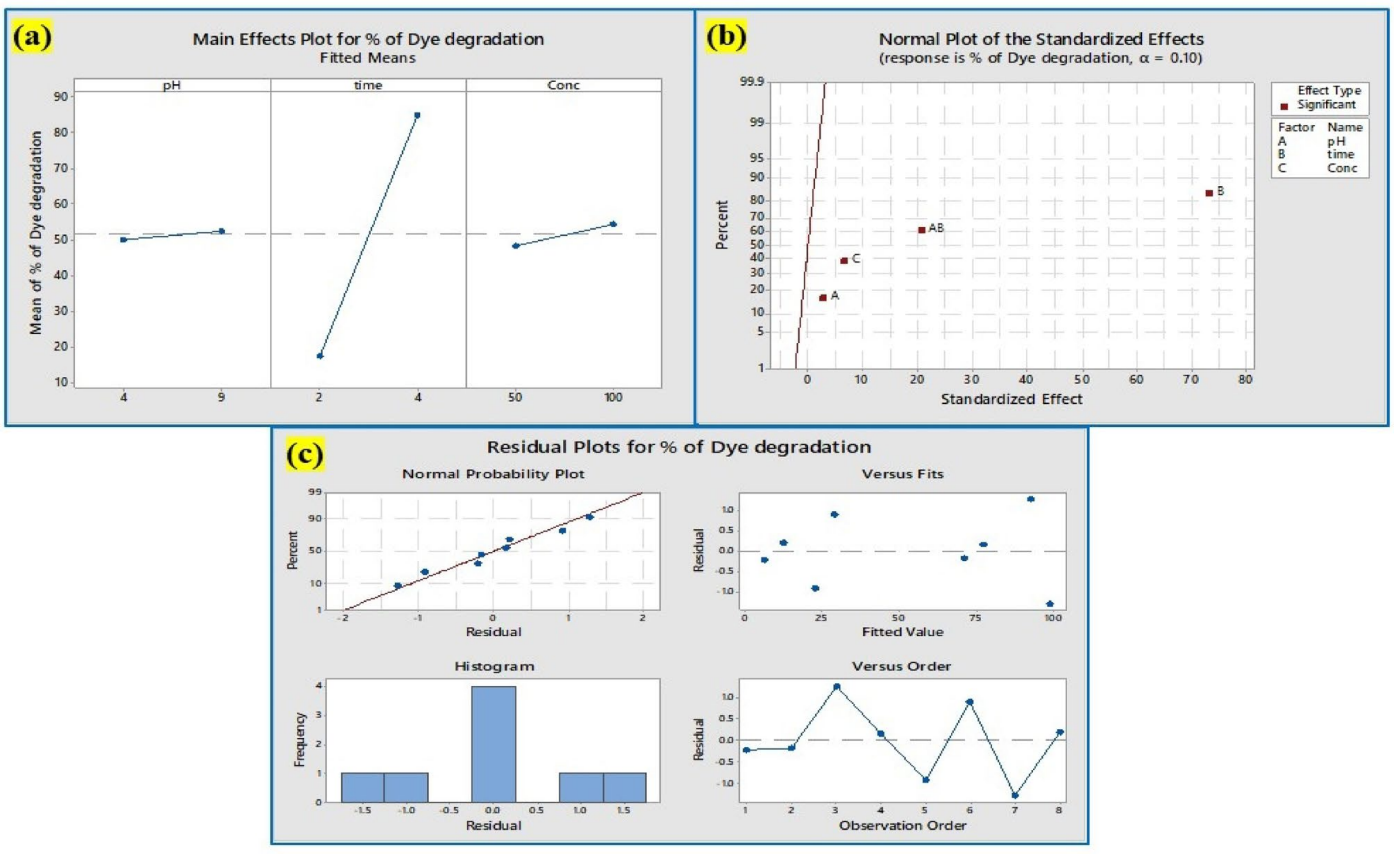
(**a**) The main plots for % of dye degradation (**b**) Normal plots of the standardized effect on % of dye degradation (**c**) The Residual plots for % of dye degradation.

**Fig. 16:**
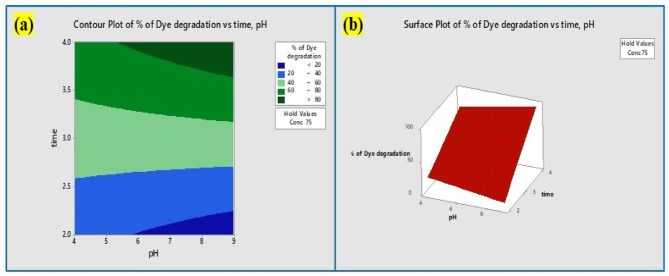
(**a**) Contour plots (**b**) Surface plot showing the effect of pH, and time on the % of dye degradation.

*Aspergillus penicillioides* arbitrated Y_2_O_3_ nanocomposites were utilized for photocatalytic degradation^46^, which yielded encouraging outcomes for a variety of parameters impacting the elimination of crystal violet dye. The association plot for the percentage of dye degradation (Fig. 14a) suggested that pH and time played a significant role, although the influence of pH versus concentration and time versus concentration on dye degradation was less apparent. The cube plot (Fig. 14b) shows the relationship between pH, time, and the concentration of the nanocomposite for the degradation. The main effect (Fig. 15a) shows that time had a greater impact on the elimination of dye, whereas the normal plot (Fig. 15b) underscored that pH and time had a greater impact on the elimination of crystal violet dye compared to concentration. Based on the results, the residual plot demonstrated that the linear model was suitable (Fig. 15c). In consonance with the Pareto chart (Fig. 14c), time and nanocomposite concentration had a greater impact on dye degradation than other variables. The counterplot (Fig. 16a) and surface plot (Fig. 16b) demonstrated that pH and time were critical factors in the elimination of crystal violet, and the equation supported the statistical relevance of dosage, time, and pH. The findings indicate that pH and time are crucial factors in the disintegration of crystal violet employing *Aspergillus penicillioides* arbitrated Y_2_O_3_ nanocomposites^32^.

#### Antibacterial study on Y_2_O_3_ nanocomposites

##### Agar well diffusion method

**Fig. 17:**
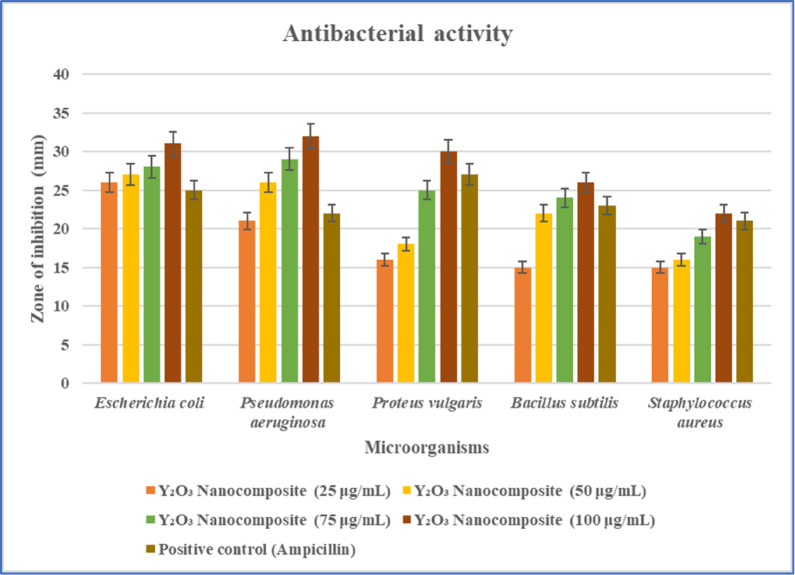
Antibacterial activity—agar well diffusion method by using myco-synthesized Y_2_O_3_ nanocomposite against gram-positive and gram-negative bacteria.

The antibacterial efficacy of the *Aspergillus penicillioides* arbitrated Y_2_O_3_ nanocomposites was evaluated against the following bacteria: *P. aeruginosa*,* E. coli*, *B. subtilis*, *P. vulgaris*, and *S. aureus.* The zone of inhibition obtained by employing nanocomposites is compared with the positive control (ampicillin). Y_2_O_3_ nanocomposites have strong antibacterial activity against *B. subtilis*, *E. coli*, and *P. aeruginosa*, where medium activity against *P. vulgaris* and *S. aureus* (Fig. 17). An electromagnetic reaction occurs on the nanocomposite surface due to the negative charge of the bacteria and the metal oxide nanocomposites, resulting in repulsive forces. This reaction leads to the generation of reactive oxygen species or the disruption of cellular components in the microorganism, resulting in cell death. Nanocomposites can interact with phosphorus- or sulfur-containing substances, such as DNA and protein thiol groups, inhibiting DNA replication and inactivating proteins may lead to damage to the bacterial cells. Moreover, nanocomposites can generate channels in bacterial cell walls, increasing cell permeability and ultimately causing cell death. Recent reports suggest that a long-lasting repulsive force between the bacterial cell walls and metal nanocomposites contributes to cell death. Due to their properties, Y_2_O_3_ nanocomposites can effectively function as antibacterial agents against microbes^47^.

##### Bacterial growth kinetics

Bacterial isolates, including *P. aeruginosa*,* E. coli*,* P. vulgaris*,* B. subtilis*, and *S. aureus*, when cultured under standard conditions, typically go through the lag, log, stationary, and decline growth phases. However, when treated with *Aspergillus penicillioides* arbitrated yttrium oxide nanocomposites, the log phase of these bacteria was notably shortened, indicating the bacteriostatic and bactericidal properties of the myco-synthesized Y_2_O_3_ nanocomposites. Distinct lag phases were observed between the test cultures treated with Y_2_O_3_ nanocomposites and the untreated control cultures. While there was no significant difference in the overall growth rates of the test and control samples for the first 7 h, a slight decrease in growth was noted in the Y_2_O_3_ nanocomposite-treated bacteria afterward. This suggests that the nanocomposites penetrated the bacterial cells, initiating antibiotic action.

**Fig. 18:**
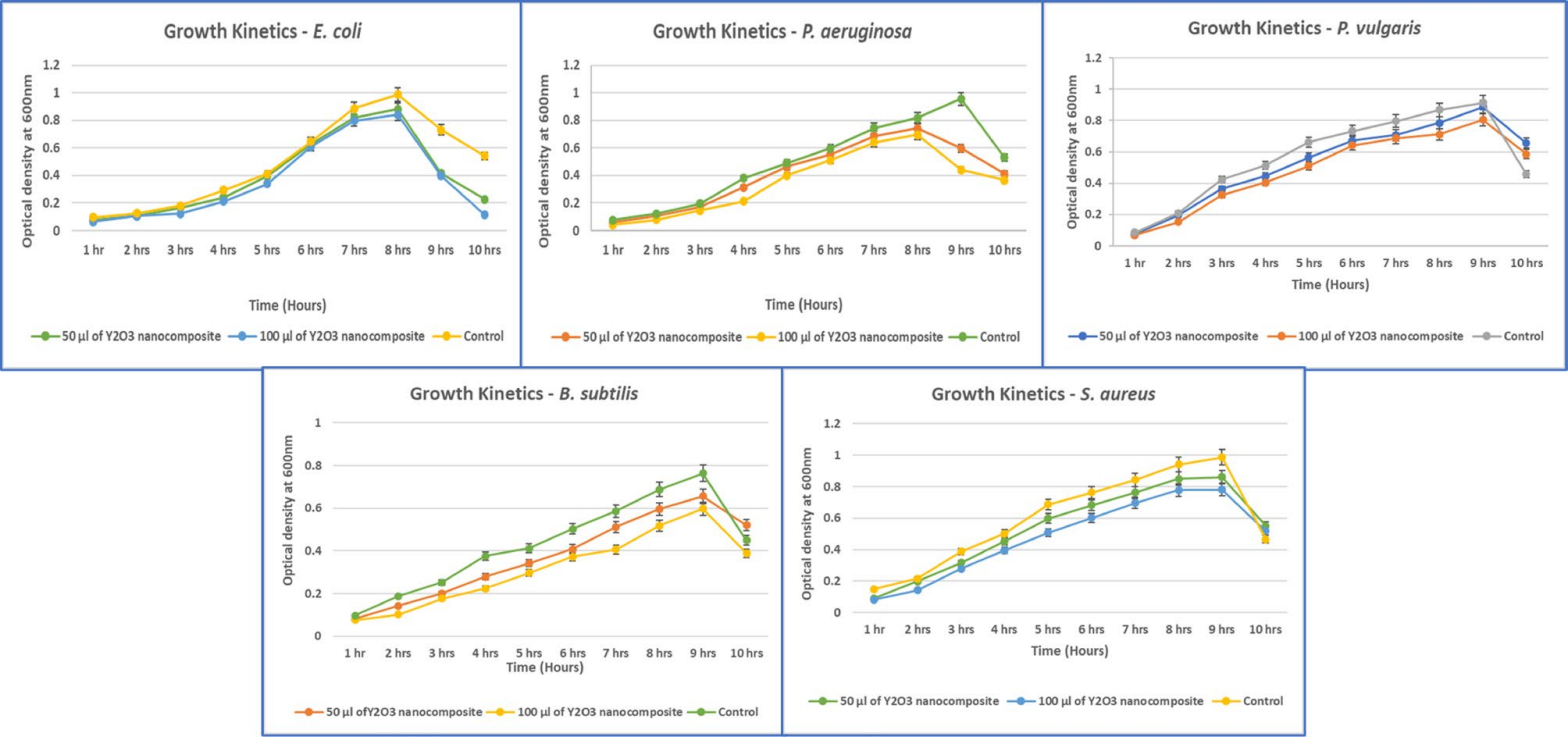
The growth curve of bacterial species treated with different concentrations of myco-synthesized Y_2_O_3_ nanocomposite (50 and 100 µg/ml) is depicted in the graphs.

Yttrium oxide nanocomposites exhibit potent antibacterial properties against all five strains (Fig. 18). These nanocomposites interact with bacterial DNA, inactivate bacterial enzymes, and create perforations in cell membranes, leading to cell death. The effectiveness of these antibacterial agents is influenced by factors such as interaction charge, concentration, shape, size, and cell type. Research has conclusively shown that nanocomposites can easily infiltrate bacterial cells, thereby inhibiting or reducing bacterial growth^48^.

##### Protein leakage analysis

**Fig. 19:**
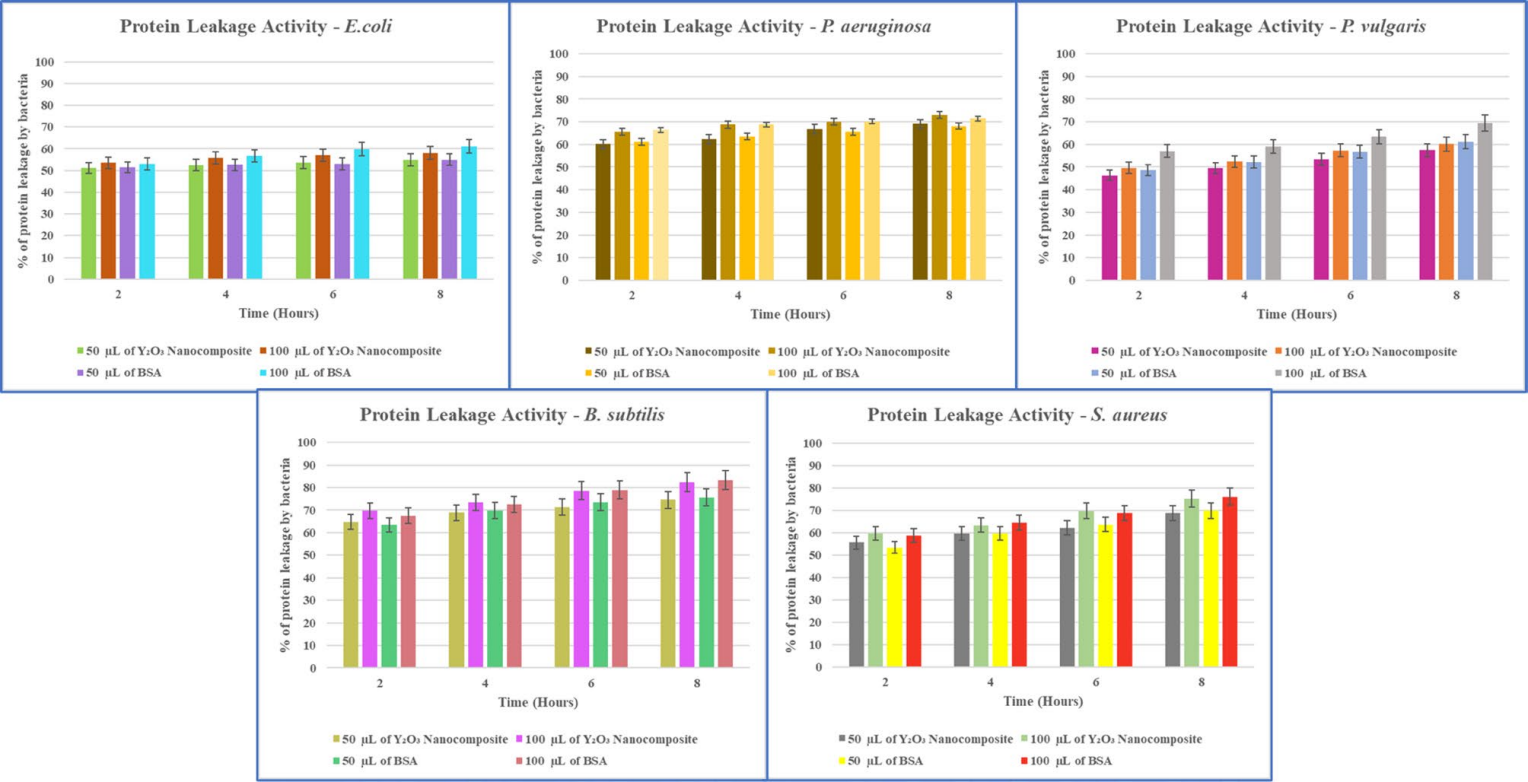
The graph illustrates the analysis of bacterial protein leakage by Bradford assay.

The solution concentration, pH, treatment duration, active oxygen formation, surface properties, particle size, and shape are several factors that have been examined to determine the antibacterial effectiveness of metallic oxide nanocomposites^49^. Figure 19 illustrates the total protein produced in the samples by the treated cells, as determined by the Bradford assay. The protein release from the bacterial cells elevated with the concentration and duration of Y_2_O_3_ nanocomposites. Notably, the size of the nanocomposites significantly impacts their antibacterial efficiency. The outer membranes of bacterial cells contain pores of nanometer dimensions, suggesting that nanocomposites can easily penetrate these membranes due to their smaller diameters than the bacterial pores^8^. Additionally, the antibacterial activity of metallic oxide nanocomposites is primarily attributed to their surface interaction with protein thiol (-SH) groups in the cell walls. This synergism reduces cell porosity and leads to cell lysis. When the cell membrane is compromised, proteins, minerals, and genetic materials quickly leak out, resulting in cell death^50^.

Bacterial strains exhibited protein leakage, suggesting that many cells exposed to nanocomposites were compromised, with intracellular materials seeping into the surrounding solution (Fig. 19). The extent of cell membrane rupture, indicated by the increased production of the enzyme lactate dehydrogenase, is dependent on the duration of exposure^36^. The research shows that the antibacterial impact of myco-synthesized Y_2_O_3_ nanocomposites was particularly detrimental to *P. aeruginosa*,* E. coli*,* B. subtilis*, and *P. vulgaris*. In contrast, *S. aureus* showed a moderate protein leakage effect when treated with varying quantities of *Aspergillus penicillioides* arbitrated Y_2_O_3_ nanocomposites.

#### Antioxidant activity: DPPH assay

**Fig. 20:**
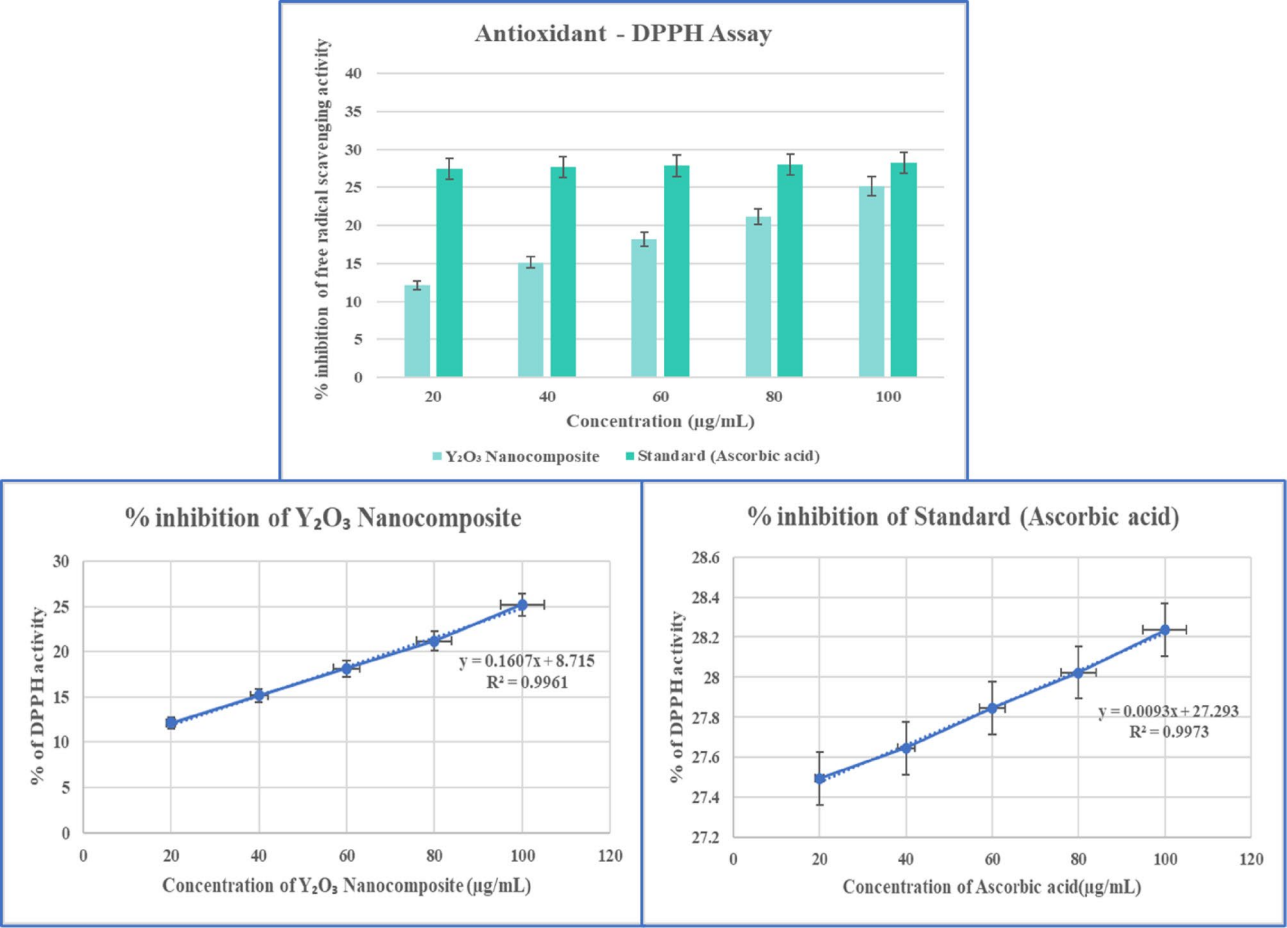
The myco-synthesized Y_2_O_3_ nanocomposite’s DPPH antioxidant inhibitory activity is depicted in the graph. The synthesized Y_2_O_3_ nanocomposite reveals a comparable level of antioxidant activity to standard ascorbic acid. The IC_50_ value was also computed using standard error, with a p-value of less than 0.05.

The 2,2-diphenylpicrylhydrazyl radical, a stable free radical, can be used to measure antioxidant activity^51^. The antioxidant potential of the *Aspergillus penicillioides* arbitrated Y_2_O_3_ nanocomposite was assessed by determining the inhibition levels of DPPH radicals and comparing them to ascorbic acid (used as a standard). The Y_2_O_3_ nanocomposite’s capacity to absorb DPPH radicals increased with its concentration. Figure 20 depicts the free radical scavenging activity of DPPH. The Y_2_O_3_ nanocomposite expressed antioxidant activity levels comparable to standard ascorbic acid. The results confirmed the effectiveness of the Y_2_O_3_ nanocomposite, derived from fungal metabolites, as a radical scavenger. Radicals serve as regulatory and signaling molecules in live cells and are naturally produced during cellular metabolic processes. Using ascorbic acid as a benchmark, the anti-oxidant IC_50_ value of the myco-synthesized Y_2_O_3_ nanocomposite was found to be 10.37 ± 0.04 µg, while the IC_50_ value for ascorbic acid was determined to be 27.29 ± 0.07 µg.

#### Hemolytic assay

**Fig. 21:**
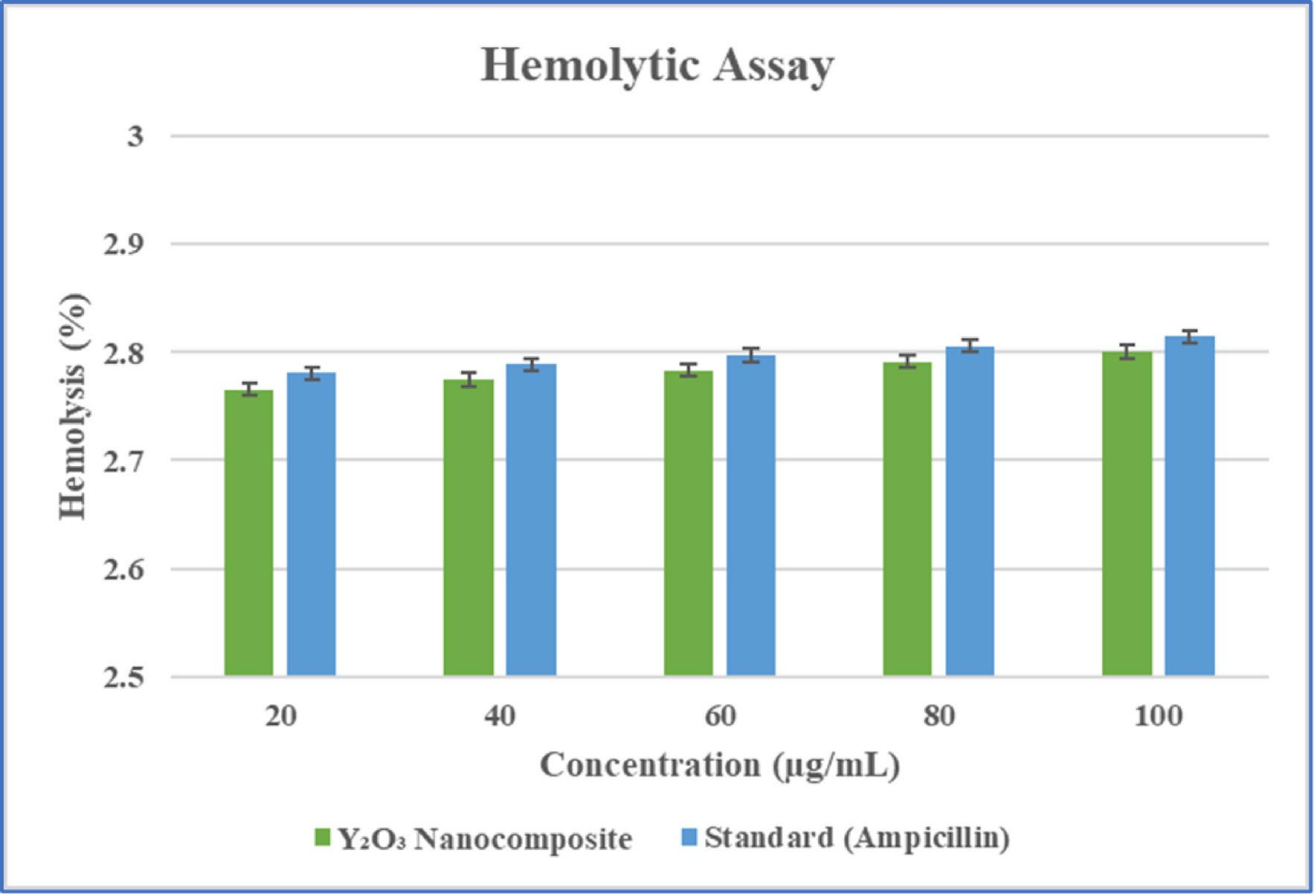
Hemolytic assay to evaluate the toxicity of Y_2_O_3_ nanocomposites.

The acute toxicity of the nanocomposites on red blood cells was determined by a hemolytic assay^38^. The percentage of Y_2_O_3_ nanocomposite samples that showed hemolysis ranged from 2.8 to 2.77%, with none of the doses showing any noticeable signs. Figure 21 shows that the Y_2_O_3_ nanocomposite performs well within acceptable ranges when compared to the standard ampicillin hemolysis percentage (2.78% to 2.82%). This finding concluded that the Y_2_O_3_ nanocomposite produced via mycosynthesis was non-hemolytic.

### Desorption and reutilization efficiency of Y_2_O_3_ nanocomposite

**Fig. 22:**
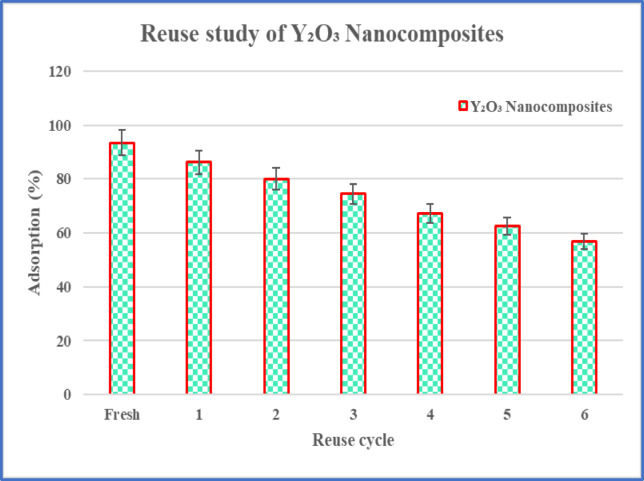
A bar graph illustrating the reuse study of Y_2_O_3_ nanocomposites, along with their corresponding adsorption percentages for each cycle.

The process of lead and nickel adsorption on the surface of a nanocomposite is reversible. After the desorption of hazardous metal ions, the nanocomposites can be reused. To extract the metal ions from the surface of the nanocomposites, 2.0 mL of a 1:1 solution of methanol and NaOH (0.1 mol/L) was employed to elute the adsorbent^39^. A multitude of cycles were conducted to evaluate the adsorbent’s reusability capacity. It was shown that after 7 cycles of adsorption and desorption, the regenerated adsorbent could eliminate over 93.4% of the targeted metal ions from the sample solution. The first cycle will eliminate 93.4% and the last 7th cycle will eliminate the metal ions up to 56.8% (Fig. 22).

### Future implications of the study

This study establishes a robust basis for the synthesis of Y_2_O_3_ nanocompositesutilizing *Aspergillus penicillioides* as a biological catalyst for the remediation of lead and nickel. This bio-mediated technique has significant promise for scaling to pilot and industrial applications, especially in the treatment of effluents from the electroplating, battery, and paint industries. To enhance the validation of the environmental compatibility and therapeutic safety of the nanocomposite, in vivo evaluations utilizing model organisms such as zebrafish, Drosophila melanogaster, or rodents can yield profound insights into biological distribution, metabolic pathways, and chronic toxicity.

Integrating real-time wastewater samples with intricate matrices will further assess the nanocomposite’s efficacy under diverse physicochemical conditions. Furthermore, integrating this technology with membrane filtration or bioreactor-based remediation units could improve practical use. Additional genetic and metabolic analysis of the fungal system may facilitate improved yield and customized nanostructures with heightened metal affinity. This bio-nanotechnological platform facilitates the development of advanced, sustainable solutions for heavy metal remediation that adhere to ecological and biomedical safety regulations.

## Conclusion

Y_2_O_3_ nanocomposites are gaining significance in wastewater remediation (lead and nickel) due to their distinctive characteristics, including adsorption capacity, photocatalytic activity, chemical stability, selective binding, biocompatibility, and versatility while minimizing the use of chemicals and energy-intensive processes. The present research determined its adsorption capability by integrating and characterizing Y_2_O_3_ nanocomposites and using an appropriate batch adsorption analysis that considers the impacts of toxic metal ion concentration, pH, dose of the nanocomposite, and irradiation source. Major focus areas for this research include the kinetic studies and the adsorption isotherm. The most significant degradation of crystal violet dye using photocatalysis is 84% in 4 h and 23% in 2 h. The proportion of lead and nickel that remained in the wastewater was further confirmed by the AAS data and the adsorption percentage was found to be 31.74 and 37.2. Additionally, Y_2_O_3_ nanocomposites exhibited exceptional antibacterial and antioxidant activity. The hemolytic assay revealed the biocompatability of the Y_2_O_3_ nanocomposite. This study shows that wastewater may be efficiently treated to remove lead, nickel, and crystal violet using catalytic Y_2_O_3_ nanocomposites.

## Supplementary Information

Below is the link to the electronic supplementary material.

Supplementary Material 1

## Data Availability

The datasets generated and/or analyzed during the current study are not publicly available due to confidentiality, but are available from the corresponding author upon reasonable request (NCBI gene sequence: [https://www.ncbi.nlm.nih.gov/nuccore/2847402264](https:/www.ncbi.nlm.nih.gov/nuccore/2847402264) ).
