# Supplementary material for: Biological applications of yttrium oxide nanocomposites synthesized from Aspergillus penicillioides and their potential role in environmental remediation
Source: Sci Rep. 2025 Oct 24;15:37211. doi: 10.1038/s41598-025-21104-4 (PMC12552491; doi:10.1038/s41598-025-21104-4)
Supplement: Supplementary file 1 — Supplementary Material 1 [file 41598_2025_21104_MOESM1_ESM.docx]

**Biological Applications of Yttrium oxide Nanocomposites synthesized from *Aspergillus penicillioides* and their Potential Role in Environmental Remediation**

**Supplementary file**

**Table. 1: EDX analysis of Y_2_O_3_ nanocomposites**

| **Element** | **Line Type** | **Wt%** | **Wt% Sigma** | **Atomic %** |
| --- | --- | --- | --- | --- |
| C | K series | 34.05 | 0.96 | 57.75 |
| O | K series | 26.00 | 0.65 | 33.10 |
| Y | L series | 39.95 | 0.70 | 9.15 |
| Total: |  | 100.00 |  | 100.00 |

**Table. 2: Adsorption Isotherm and kinetic parameters**

| **Adsorption model** | **Equation** | **Plot** | **Parameters** | | **R^2^** | |
| --- | --- | --- | --- | --- | --- | --- |
|  |  |  | **Lead** | **Nickel** | **Lead** | **Nickel** |
| Langmuir adsorption isotherm | C_e_ / q_e_ = C_e_ / q_m_ + (1/kq_m_) | C_e_ Vs C_e_/q_e_ | q_m_ = 1.58 | q_m_ = 4.73 | 0.97982 | 0.97982 |
|  |  |  | KL = 0.09 | KL = 0.09 |  |  |
| Freundlich adsorption isotherm | Log q_e_ = (1/n) log C_e_ + log k | log C_e_ vs log q_e_ | 1/n = 1.40 | 1/n = 1.40 | 0.99406 | 0.99406 |
|  |  |  | K_f_ = 0.15 | K_f_ = 0.46 |  |  |
| Pseudo-first-order kinetic model | Log (q_e_-q_t_) = log (q_e_) – (k/2.303)t | t vs log(q_e_-q_t_) | q_e_ = 1.64 | q_e_ = 0.84 | 0.28332 | 0.3732 |
|  |  |  | K_1_ = 0 | K_1_ = 0 |  |  |
| Pseudo-second-order kinetic model | t/q_t_ = 1/kq_e_^2^ + t/q_e_ | t vs t/q_t_ | q_e_ = 1.03 | q_e_ = 3.11 | 0.99999 | 0.99999 |
|  |  |  | K_2_ = 3 | K_2_ = 1 |  |  |

**Table. 3: The analysis of variance (ANOVA) for dye degradation**

| **Source** | **DF** | **Adj SS** | **Adj MS** | **F-Value** | **P-Value** |
| --- | --- | --- | --- | --- | --- |
| **Model** | 4 | 9950.85 | 2487.71 | 1456.58 | 0.000 |
| **Linear** | 3 | 9219.32 | 3073.11 | 1799.33 | 0.000 |
| **pH** | 1 | 12.75 | 12.75 | 7.47 | 0.072 |
| **Time** | 1 | 9132.76 | 9132.76 | 5347.31 | 0.000 |
| **Concentration** | 1 | 73.81 | 73.81 | 43.22 | 0.007 |
| **2-way Interaction** | 1 | 731.53 | 731.53 | 428.32 | 0.000 |
| **pH*time** | 1 | 731.53 | 731.53 | 428.32 | 0.000 |
| **Error** | 3 | 5.12 | 1.71 | 0 | 0 |
| **Total** | 7 | 9955.98 | 0 | 0 | 0 |

**Model Summary**

| **S** | **R-sq** | **R-sq(adj)** | **R-sq(pred)** |
| --- | --- | --- | --- |
| 1.30687 | 99.95% | 99.88% | 99.63% |

* Values are statistically significant at p ˃ 0.05, so concentration, pH, and time affect the dye degradation when used together.

**Table. 4: Regression equation for dye degradation**

**Coded Coefficients**

| **Term** | **Effect** | **Coef** | **SE Coef** | **T-Value** | **p-value** | **VIF** |
| --- | --- | --- | --- | --- | --- | --- |
| **Constant** | 0 | 51.337 | 0.462 | 111.11 | 0.000 | 0 |
| **pH** | 2.525 | 1.263 | 0.462 | 2.73 | 0.072 | 1.00 |
| **Time** | 67.575 | 33.788 | 0.462 | 73.13 | 0.000 | 1.00 |
| **Concentration** | 6.075 | 3.038 | 0.462 | 6.57 | 0.007 | 1.00 |
| **pH* Concentration** | 19.125 | 9.562 | 0.462 | 20.70 | 0.000 | 1.00 |

Regression Equation in Uncoded Units

% of dye degradation = 12.17 – 10.970 pH + 8.93 time + 0.1215 Concentration + 3.825 pH*time

* Values are statistically significant at p ˃ 0.05 as pH, time, and concentration affect the dye degradation.

**Table. 5: Antibacterial activity of Y_2_O_3_ nanocomposite**

| **Microbes** | **Positive control (mm)** | **Y_2_O_3_ nanocomposite (mm)** | | | |
| --- | --- | --- | --- | --- | --- |
|  |  | **(25 μg/mL)** | **(50 μg/mL)** | **(75 μg/mL)** | **(100 μg/mL)** |
| *Escherichia coli* | 25 ± 0.43 | 26 ± 0.32 | 27 ± 0.12 | 28 ± 0.43 | 31 ± 0.37 |
| *Pseudomonas aeruginosa* | 22 ± 0.45 | 21 ± 0.32 | 26 ± 0.12 | 29 ± 0.14 | 32 ± 0.20 |
| *Proteus vulgaris* | 27 ± 0.24 | 16 ± 0.32 | 18 ± 0.31 | 25 ± 0.41 | 30 ± 0.16 |
| *Bacillus subtilis* | 23 ± 0.54 | 15 ± 0.04 | 22 ± 0.13 | 24 ± 0.20 | 26 ± 0.33 |
| *Staphylococcus aureus* | 21 ± 0.55 | 15 ± 0.22 | 16 ± 0.41 | 19 ± 0.15 | 22 ± 0.12 |
